# High-Density Lipoproteins: A Role in Inflammation in COPD

**DOI:** 10.3390/ijms23158128

**Published:** 2022-07-23

**Authors:** Stanislav Kotlyarov

**Affiliations:** Department of Nursing, Ryazan State Medical University, 390026 Ryazan, Russia; skmr1@yandex.ru

**Keywords:** HDL, lipoproteins, apolipoproteins, COPD, innate immune system, inflammation, ABCA1, ApoA-I

## Abstract

Chronic obstructive pulmonary disease (COPD) is a widespread disease associated with high rates of disability and mortality. COPD is characterized by chronic inflammation in the bronchi as well as systemic inflammation, which contributes significantly to the clinically heterogeneous course of the disease. Lipid metabolism disorders are common in COPD, being a part of its pathogenesis. High-density lipoproteins (HDLs) are not only involved in lipid metabolism, but are also part of the organism’s immune and antioxidant defense. In addition, HDL is a versatile transport system for endogenous regulatory agents and is also involved in the removal of exogenous substances such as lipopolysaccharide. These functions, as well as information about lipoprotein metabolism disorders in COPD, allow a broader assessment of their role in the pathogenesis of heterogeneous and comorbid course of the disease.

## 1. Introduction

Chronic obstructive pulmonary disease (COPD) is a widespread socially significant disease [1,2]. It leads to a large number of cases of seeking medical care and temporary and permanent disability every year in the world [3,4]. Moreover, COPD occupies one of the leading positions in the structure of mortality causes, which undoubtedly emphasizes the urgency of the problem [5,6].

COPD is characterized by a progressive decline in lung function, with chronic respiratory symptoms such as cough, sputum production, and dyspnea [7,8]. The severity of symptoms can be variable, up to and including significant limitation of physical activity as a result of dyspnea [9,10]. In addition, COPD is characterized by the development of physical frailty, mainly owing to skeletal muscle dysfunction of the lower extremities, which further reduces the quality of life of these patients [11,12].

A key etiologic factor in the development of COPD is long-term exposure to components of tobacco smoke [13]. It is believed that COPD develops in susceptible smokers, and smoking cessation can reduce the rate of decline in lung function, which is seen as an effective therapeutic tool for all stages of COPD [14,15,16]. Early smoking initiation may be additional factor influencing the risk of COPD [17]. Exposure to inhaled particulate matter is considered another significant cause of COPD [18]. This is also a relevant problem, given the negative air quality data in large cities [19,20]. The genetic factor also plays an important role in the development of the disease, which is well known to clinicians by the presence of alpha 1-antitrypsin deficiency phenotype [21].

According to modern concepts, COPD is a disease whose pathophysiological basis is chronic airway inflammation, which develops in response to long-term exposure to tobacco smoke components [22,23]. Many cells involved in this inflammation, including macrophages and neutrophils, are part of the innate immune defense of the lungs [24,25,26]. The mechanisms of the innate immune system have been shown to be impaired in smoking and COPD [27]. This seems important given that large volumes of inhaled air, containing many different particles and microorganisms, pass through the lungs every day. Impairments in the mechanisms of innate immune protection of the lungs lead to changes in the structure of the airway microbiome, which negatively affect the progression of the disease, including at the expense of infectious exacerbations [28,29]. Infectious exacerbations of COPD increase the reduction of lung function and contribute to systemic inflammation [30,31,32]. Thus, the imbalance in inflammation control mechanisms leads to its chronicity and the possibility of systemic inflammation, which may act as a link between COPD and a number of comorbid diseases [33,34].

A growing body of evidence is paying increasing attention to the problem of metabolic abnormalities in COPD. Studies in the pathophysiology of COPD show many cross-links between inflammation and lipid metabolism disorders [35]. These links are increasingly becoming the subject of close attention of researchers and clinicians when studying the comorbid course of COPD with cardiovascular disease [36,37]. Metabolic syndrome and diseases associated with atherosclerosis of different localizations are often found in patients with COPD and are one of the key causes of hospitalization and death in these patients.

The pathogenesis of cardiovascular disease is closely related to the involvement of lipoproteins, which is supported by the results of numerous studies [38,39,40,41]. The plasma cholesterol level is a reliable marker that is widely used for the diagnosis and prediction of cardiovascular risk [42]. Pharmacological correction of lipid profile parameters is a well-known therapeutic strategy that has shown its effectiveness [41]. At the same time, there is increasing evidence that lipoprotein function may be associated with the development and progression of COPD [43,44,45,46]. Patients with severe and very severe stages of COPD have been shown to have high low-density lipoprotein cholesterol (LDL) and low high-density lipoprotein (HDL) blood levels [43].

HDL levels are an important indicator in the prognostic model of cardiovascular risk in patients with COPD [47]. These and other data increase attention to HDL as an antiatherogenic particle and as an anti-inflammatory agent that may be involved in the pathogenesis not only of cardiovascular disease, but also of COPD.

Thus, the purpose of this review is to discuss the involvement of HDLs in inflammation and their possible role in the development of COPD.

## 2. Clinical Links between HDL and COPD

**Figure 1:**
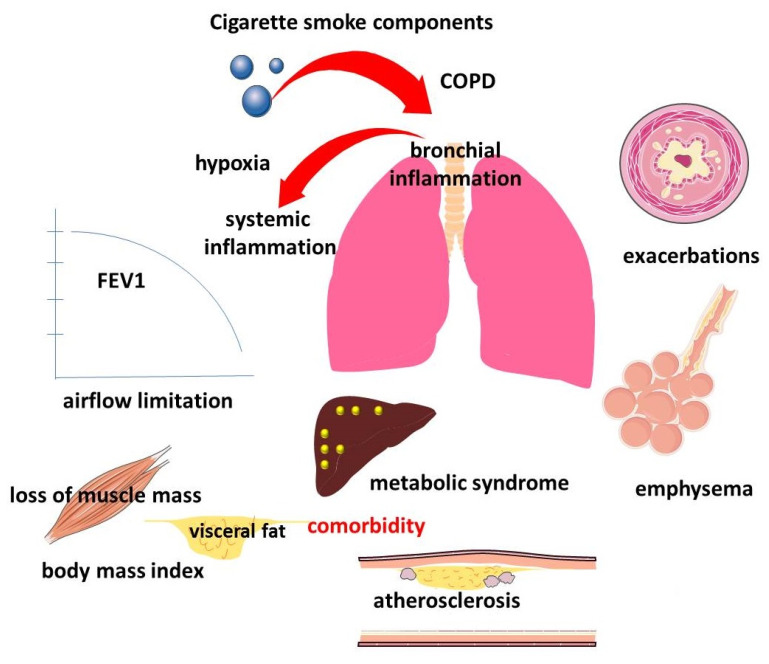
Manifestations of pulmonary and extrapulmonary clinical heterogeneity of COPD.

According to modern concepts, COPD is a clinically heterogeneous disease. This clinical heterogeneity has both pulmonary and extrapulmonary manifestations, which form individual trajectories of the natural history of the disease (Figure 1). COPD is characterized by a progressive decline in lung function, which underlies the diagnosis and stratification of disease severity and prognosis [48,49]. The evidence of an association between declining lung function and HDL levels is interesting, but contradictory.

Data from an analysis of a large sample of patients over a 7-year follow-up period showed that higher levels of HDL cholesterol corresponded to a faster rate of decline in forced expiratory volume in the first second (FEV1) and FEV1/FVC (forced vital capacity). This rate of decline in magnitude of effect was similar to the 10-year increase in pack-years index [50]. Another study found that higher HDL cholesterol levels may be associated with decreased lung function (FVC and FEV1) in healthy male adolescents [51]. Another study also showed a negative correlation between HDL levels and lung function. In this study, patients with low HDL levels had better spirometric results (FEV1, FVC, and FEV1/FVC ratio) than patients with normal HDL levels [52].

Serum HDL levels were also shown to be elevated in patients with an FEV1 less than 30% [53]. Elevated HDL cholesterol levels in severe COPD may be partly related to the intake of oral steroids [54]. Another study showed that the best predictor of lung function in patients with COPD may be the ratio of lymphocytes to HDL [55]. At the same time, other studies have noted a decrease in HDL levels in COPD patients with more severe stages [43,56,57,58]. Such differences may be related to the heterogeneity of COPD itself, which is rarely considered in studies. In this regard, studies evaluating the dependence of lung function on HDL levels should be further developed, as they do not allow making unequivocal conclusions.

HDL levels have been shown to decrease in patients with COPD who have undergone lung transplantation [53]. A decreased HDL level is not associated with changes in low body mass index (BMI) and diabetes mellitus. Interestingly, such changes are only characteristic of COPD and do not occur in patients who have undergone lung transplantation for other reasons [53].

In addition to decreased lung function, another important characteristic of COPD, reflecting the clinical heterogeneity of the disease, is the development of emphysema. There is a lot of evidence supporting different mechanisms of emphysema development. Interestingly, there has been a reported association of high HDL levels with the progression of emphysema [46,51,56,59]. The MESA Lung Study showed a 0.4% increased risk of emphysema for every 10 mg/dL increase in HDL cholesterol [59]. Importantly, emphysema is a frequent COPD phenotype that has strong associations with nutritional deficiencies [60,61]. In the Japanese COPD patient population, which is dominated by patients with lower body mass index and emphysema, the prevalence of cardiovascular disease and metabolic syndrome is relatively lower compared with patients from Western countries [62].

Another important clinical characteristic of COPD is related to the frequency and severity of infectious exacerbations [63]. In a study in men, higher levels of HDL and large subfractions were associated with a higher frequency of severe exacerbations of chronic respiratory disease regardless of FEV1 [64]. A U-shaped relationship of HDL and risk of infectious disease was shown, with both lower and higher levels of HDL associated with infectious disease [65].

Comorbidity is another important clinical characteristic of the heterogeneous course of COPD [66,67]. Moreover, lower serum HDL levels in patients with COPD compared with nonsmoking individuals may partially explain the increased cardiovascular risk in these patients [44]. This seems important given the high prevalence of comorbid course of COPD and atherosclerotic cardiovascular disease. Indeed, coronary heart disease and peripheral artery disease are common in COPD patients [68,69]. Such comorbidity mutually worsens the course of both diseases. COPD and atherosclerosis are thought to have some common pathogenesis links, including those related to inflammation [70]. COPD may predispose patients to a diffuse atherosclerotic phenotype with worse clinical outcomes [68,71].

Obstructive sleep apnea in patients with COPD is associated with an even higher prevalence of metabolic syndrome [72]. This is important given the development of hypoxia caused by both COPD and obstructive sleep apnea syndrome [73,74]. In turn, metabolic syndrome is associated with a high risk of exacerbations and hospitalizations in patients with COPD [75]. Impaired lipid profile, glucose metabolism, and high blood pressure can exacerbate overall morbidity and disease outcome [76]. Importantly, COPD patients with metabolic syndrome are physically less active and have increased levels of systemic inflammation compared with COPD patients without metabolic syndrome [37]. Smoking has been shown to be an important risk factor for metabolic syndrome [77].

The evidence of the relationship between changes in body weight and COPD course is of particular interest [78,79,80]. It has been shown that BMI is associated with a worse prognosis in COPD, which is due to the negative impact of a decrease not only in adipose tissue volume, but also in skeletal muscle mass [81,82,83]. In contrast, some evidence suggests a better prognosis with excess body weight [84]. This phenomenon, termed the “obesity paradox”, remains the subject of research and is an additional factor increasing attention to lipid metabolism [85,86,87].

HDLs play an important role in the prevention of atherosclerosis, and a decrease in their levels is regarded as one of the negative markers [88]. The monocyte to HDL cholesterol ratio (MHR) has been shown to be another important indicator of inflammation and may predict cardiovascular disease in different patient groups [89,90,91,92]. MHR is associated with overall and cardiovascular mortality in the general population [93]. Elevated MHR is a marker reflecting endothelial dysfunction and the severity of systemic inflammation, which has been shown in patients with Behcet’s disease [94]. In addition, MHR can be used as a prognostic marker of cardiovascular disease in patients with COPD [92]. It has been shown that MHR levels were significantly higher in smokers than in nonsmokers. In addition, the relationship between MHR and the number of cigarettes smoked per day was established [95].

These and other data have attracted the attention of clinicians to the possibility of using statins, which have demonstrated an anti-inflammatory pleiotropic effect in a number of studies [96,97,98,99]. However, to date, there is no unequivocal answer on the effectiveness of statins in COPD patients. The use of statins is characterized by different effects on mortality rates and on exacerbations in COPD [97,98,100,101,102,103,104,105]. However, the prescription of statins has a justified value in many clinical situations, given the frequent combination of COPD with cardiovascular diseases of atherosclerotic genesis. It has been suggested that COPD may enhance the development and progression of atherosclerosis through systemic inflammation and oxidative stress. Thus, the course of COPD is characterized by clinical heterogeneity, which is not always considered in studies.

Thus, interest in the role of HDL in patients with COPD is due to several factors. First of all, they concern the comorbid course of COPD and cardiovascular diseases, in which the correction of lipid profile parameters is one of the key therapeutic strategies [106]. On the other hand, HDLs demonstrate a variety of functions in inflammation, which seems important, given the inflammatory nature of COPD and the significance of infectious exacerbations of COPD on the course of the disease and prognosis.

## 3. Anti-Inflammatory Function of HDL

HDL particles are heterogeneous in their composition, physicochemical properties, and physiological functions [107]. Mature HDLs contain a non-polar lipid core consisting mainly of cholesterol esters and triglycerides, surrounded by a surface monolayer of phospholipids, unesterified cholesterol, and apolipoproteins [107].

**Figure 2:**
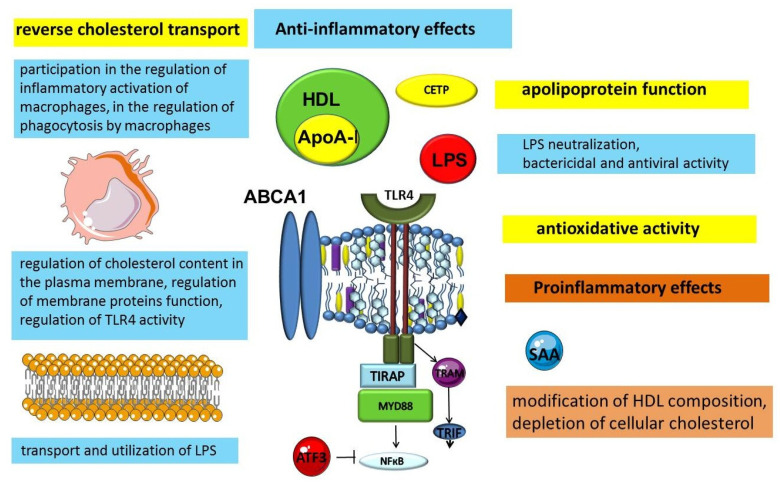
Anti-inflammatory and pro inflammatory functions of HDLs. Abbreviations: ABCA1—ATP binding cassette subfamily A member 1; ATF3—activating transcription factor 3; CETP—cholesteryl ester transfer protein; HDL—high-density lipoprotein; LPS—lipopolysaccharide; MYD88—myeloid differentiation primary response 88; NF-κB—nuclear factor-κB; SAA—serum amyloid A; TIRAP—toll-interleukin 1 receptor (TIR) domain containing adaptor protein; TLR4—toll-like receptor 4; TRAM—TRIF-related adaptor molecule; TRIF—toll/IL-1 receptor (TIR)-domain-containing adaptor inducing IFN-beta.

The most studied function of HDL is the transport of excess cholesterol from peripheral tissues to the liver, which allows them to be considered part of an important atheroprotective mechanism that provides protection against atherosclerosis (Figure 2) [108,109]. This function is part of the mechanism that ensures tissue lipid homeostasis. In turn, the lungs are an organ with a unique lipid biology that is closely related to their function. Lipid homeostasis and thus reverse cholesterol transport are essential for normal lung cell function, especially alveolar macrophages and type II pneumocytes [110,111,112]. Cellular cholesterol content is important for the inflammatory activation of alveolar macrophages [113]. At the same time, type II pneumocytes carry out the production of pulmonary surfactant, which provides optimal surface tension of alveoli, which is necessary for breathing. Surfactant is a multicomponent structure, which includes phospholipids, cholesterol, and proteins [110]. HDLs stimulate type II alveolar cells to secrete surfactant [114]. In addition, HDL is the main source of vitamin E for type II pneumocytes, because of the presence of the SR-B1 receptor on these cells [115].

A growing body of evidence is expanding the understanding of HDLs as particles that exhibit protective, including anti-inflammatory, properties (Figure 2). Lipoproteins are an evolutionarily ancient transport mechanism for lipids. The need for a specific transport system for lipid transport in the blood is related to the physicochemical properties of lipids, in particular their insolubility in water. Research results indicate that this important transport function demonstrates cross-links between metabolism and immunity. Thus, the evidence available to date suggests that lipoproteins can be considered not only as lipid transporters, but also as participants in the innate immune system [116,117,118].

### 3.1. The Significance of Apolipoproteins in the Anti-Inflammatory Function of HDLs

**Figure 3:**
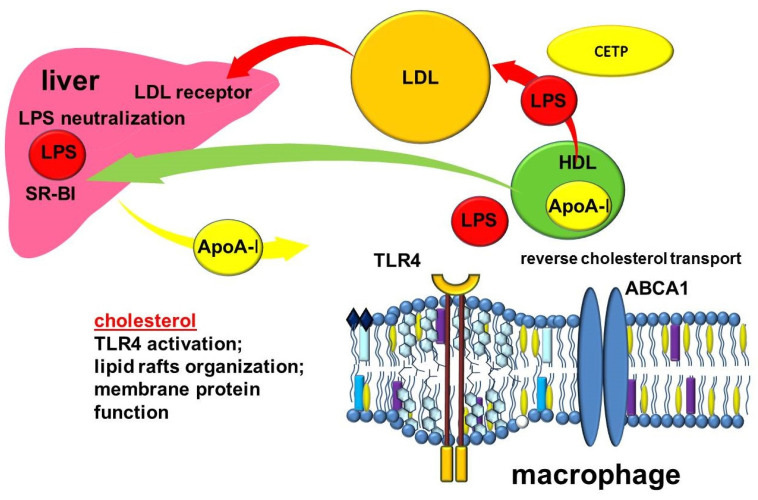
Cross-links of reverse cholesterol transport and inflammation. Abbreviations: ABCA1—ATP binding cassette subfamily A member 1; CETP—cholesteryl ester transfer protein; HDL—high-density lipoprotein; LDL—low density lipoprotein; LPS—lipopolysaccharide; TLR4—toll-like receptor 4; SR-BI—scavenger receptor class B type I.

The formation and function of HDLs depend largely on apolipoproteins, the protein components of these particles. The most abundant apolipoprotein in HDL is apolipoprotein A-I (ApoA-I), which is produced by the liver and intestine [119]. Interacting with the ATP-binding cassette sub-family A member 1 (ABCA1) transporter on the plasma membrane of cells, apoA1 takes up non-esterified cholesterol and phospholipids to form discoidal nascent HDL [117,120,121]. The transfer of cholesterol from the cells to ApoA-I to form HDL is part of a process known as reverse cholesterol transport (Figure 3). Reverse cholesterol transport removes excess cholesterol from peripheral tissues. This is a very important function that allows many clinicians to consider HDL as the so-called “good cholesterol”. The molecular mechanisms of cholesterol export by ABCA1 are the subject of much research and debate. ApoA-I increases the content of ABCA1 protein. In turn, ABCA1 can stabilize ApoA-I [122]. ApoA-I promotes ABCA1-dependent cholesterol efflux in part by inducing ABCA1 posttranslational regulation as well as through the myeloid differentiation primary response protein MyD88 (Myd88) pathway [123,124]. This suggests the involvement of ApoA-I and Myd88 macrophages in the cross-talk between innate immunity and reverse cholesterol transport [123]. In addition, Apo-AI activates lecithin-cholesterol acyltransferase (LCAT), which is involved in the maturation of HDL particles, converting free cholesterol into cholesteryl ester, which is then sequestered into the HDL core [125,126]. The subsequent interaction of lipidized ApoA-I in discoid or more mature HDL particles with another transporter, ABCG1, further contributes to reversing cholesterol transport [127,128,129].

Although ApoA-I is synthesized primarily in the liver, it is also expressed in lung cells [130]. In the adult lung, ApoA-I is expressed by epithelial cells and alveolar macrophages [131]. ApoA-I has multiple protective functions in the lung [132]. Furthermore, in the mouse fetus, ApoA-I is expressed in the lungs at higher levels than in adult mice, suggesting a role of ApoA-I in lung development [133].

ApoA1 levels were shown to be significantly reduced in the lungs of COPD patients and in the lungs of mice exposed to cigarette smoke [134]. Smokers were also found to have lower levels of HDL cholesterol and ApoA1 in the blood and higher levels of triglycerides and ApoB than nonsmokers [135]. ApoA1 overexpression in mice attenuated cigarette-smoke-induced lung inflammation and emphysema [134]. In contrast, apoA-1-deficient mice showed neutrophilia in the lungs when exposed to cigarette smoke, larger macrophages were found, and there was greater loss of muscle mass. The same study also showed that exposure to cigarette smoke activates lipid export mechanisms into more mature HDL via ABCG1 and SR-B1 in the lungs, which is insufficient to prevent accumulation of intracellular lipids by alveolar macrophages [136]. These abnormalities correspond to a decrease in reverse lipid transport to about 50% of that in wild-type mice. An exacerbated immune response to cigarette smoke exposure was also noted [136]. In addition to the described changes, genetic loss of ApoA-I in mice increased airway hyperresponsiveness, collagen deposition, and levels of oxidative stress biomarkers in the lungs [132]. In turn, clinical data showed that serum ApoA-I and large HDL particles were positively correlated with FEV1 in atopic asthma [137].

When analyzing the experimental data, it is necessary to consider the differences in the structure of lipoproteins in mice and humans, in particular the ratio of HDL to low-density lipoproteins (LDL) [138].

Thus, apoA-1, being a key participant in the lipid-transport function of HDL, is actively involved in lung function and cross-links with the innate immune system. In turn, HDL, in addition to lipid transport, can exert both anti-inflammatory and proinflammatory effects, which is determined by their composition, participation in the maintenance of cellular cholesterol composition, and involvement in some signaling pathways.

The function of ApoA-I/HDL in innate immunity has deep evolutionary roots [139]. The involvement of ApoA-I in immune defense is related to its ability to bind and neutralize the lipopolysaccharide (LPS) of Gram-negative bacteria as well as lipoteichoic acid (LTA) of Gram-positive bacteria [140,141,142,143,144,145]. The domains that mediate the neutralization of the endotoxic effect of lipopolysaccharides are located in the C-terminal of ApoA-I [143]. The results of biophysical measurements suggest that ApoA-I binds to the lipid A backbone, in particular to the diglucosamine-phosphate region of endotoxin [146]. This function of ApoA-I may represent an important mechanism of the body’s defense against Gram-negative septic shock [147,148]. The LPS-binding protein (LBP), which transfers LPS not only to CD14, but also to HDL particles, is involved in the binding and neutralization of LPS [149]. In addition, ApoA-I has antibacterial activity against some bacteria, which has been shown in both mammals and various fish species [150,151,152]. For example, BbApoA-I in amphioxus showed antibacterial activity in vitro against Gram-negative bacteria. Moreover, the expression of BbApoA-I increased after lipopolysaccharide administration [153]. The C-terminal domain of ApoA-I is an effector site providing bactericidal activity [154].

In addition to antibacterial activity, ApoA-I also has antiviral activity against herpes simplex virus and human immunodeficiency virus. The antiviral activity of ApoA-I is associated with the prevention of viral entry into cells [155].

ApoA-I can also induce anti-inflammatory signaling in macrophages through ABCA1 by activating STAT3-SOCS signaling in addition to the anti-inflammatory effect of cholesterol efflux [156].

In addition, while palmitic acid was shown to activate NF-κB signaling through toll-like receptor (TLR) 4 recruitment to lipid rafts, ApoA-I attenuated palmitate-induced NF-κB activation. In endothelial cells, ApoA-I prevents palmitate-induced TLR4 transport into lipid rafts, thereby blocking NF-κB activation [157].

Apolipoprotein A-1 binding protein (AIBP) promotes ApoA-1 binding to ABCA1 and prevents CSN2-mediated degradation of ABCA1 protein [158]. AIBP is associated with the stimulation of cholesterol efflux, resulting in the depletion of membrane cholesterol, which is essential for lipid raft integrity. Another mechanism of lipid raft modulation associated with AIBP includes AIBP interaction with PI(3)P, activation of Cdc42, and modification of the cytoskeleton [159].

These mechanisms may act as a link in the regulation of inflammation. Indeed, in addition to increasing cholesterol efflux to HDL, AIBP reduces inflammation in the lungs. AIBP reduces LPS-mediated inflammatory activation of macrophages. It inhibits inflammatory signaling pathways by binding ApoA-1 and stabilizing ABCA1, with subsequent changes in lipid rafts and TLR4 in the plasma membrane [160]. In mice subjected to LPS inhalation, AIBP was expressed in inflammatory cells in the lungs and secreted into the bronchoalveolar space. It has been shown that, through this mechanism, AIBP can increase cholesterol efflux from alveolar macrophages to the surfactant and reduce acute lung inflammation [161].

Apolipoprotein A-II (Apo-AII) is the second most abundant protein in HDL particles. Apo-AII demonstrates a proinflammatory role by enhancing the response of monocytes to LPS by suppressing the inhibitory activity of LPS-binding protein. In addition, apolipoproteins Apo-AI and Apo-AII have been shown to be involved in the regulation of neutrophil activity. ApoA-I affects the release of interleukin (IL)-1β, and Apo-AII affects the release of IL-8 from LPS-stimulated neutrophils [162,163].

Apolipoprotein M (ApoM), another protein component of HDL, may also be related to lung function [46,51,56]. Serum ApoM has been shown to be elevated in patients with COPD and to increase progressively with COPD severity, but was not associated with coronary heart disease (CHD) in patients with COPD [56].

Thus, apolipoproteins, being an important structural and functional component of HDL, are at the intersection of cholesterol export pathways and innate immune system mechanisms. At the same time, their clinical significance for COPD is the subject of debate and active research.

### 3.2. The Importance of ABC-Mediated Lipid Transport in the Regulation of Inflammation

Reverse cholesterol transport depends on the function of ABCA1 and ATP-binding cassette sub-family G member 1 (ABCG1), which are transport proteins that ensure the export of cellular cholesterol to forming HDLs [164].

ABCA1 is a key participant in the export of cholesterol from macrophages and, through this function, the transporter may be involved in the regulation of inflammation [165]. Involvement in inflammation may be realized through several known mechanisms [36]. A decrease in the functional activity of ABCA1 leads to an increase in the cholesterol content of the plasma membrane, which may affect its biophysical properties and function. In addition, it leads to cellular accumulation of cholesterol in macrophages with the formation of “foam cells” [166]. These processes contribute to the inflammatory activation of macrophages [167,168]. In addition, ABCA1 may be involved in the regulation of phagocytosis by macrophages [169]. This is because ABCA1 enhances reverse cholesterol transport in phagocytic-active macrophages, reducing the cholesterol load after uptake by apoptotic cells. In addition, another member of the ABC family of transporters, ABCA7, which is a homologue of ABCA1, may be a key molecule for linking cellular cholesterol homeostasis and the host defense system [170,171]. ApoA-I has been shown to stabilize ABCA7 and enhance phagocytosis [172].

It is suggested that the lipid-transporting activity of ABCA1 is essential for normal lung function [165]. In experiments with ABCA1 knockout mice, marked changes were found in the lungs that included alveolar proteinosis, thickening of the interalveolar septa, hyperplasia of type II pneumocytes, and formation of foamy alveolar macrophages [173]. These changes intensified with age and were accompanied by disruption of alveolar architecture and epithelialization of the remaining alveoli as a result of hyperplasia of type II pneumocytes [174]. These studies showed decreased expression of ABCA1 in lung tissues in smoking and COPD patients [175].

It has been shown that HDL increases the expression of ABCA1 and can activate the p38MAPK signaling pathway, which promotes the migration and proliferation of alveolar type II epithelial cells stimulated by LPS. In addition, HDL inhibits the secretion of pro-inflammatory TNF-a and IL-1a and IL-6 [176].

ABCG1, like ABCA1, exports cholesterol from peripheral cells, saturating HDL with it and protecting cells from sterol overload [177,178,179]. At the same time, ABCA1 performs primary cholesterol saturation of lipid-poor apoA-I, forming nascent HDL, while ABCG1 performs further cholesterol loading of more mature HDL [180,181,182,183,184]. ABCG1 is expressed in various types of lung cells, such as alveolar macrophages and epithelial cells, including type II pneumocytes [165,185]. The absence of ABCG1 leads to impaired regulation of intracellular cholesterol levels and progressive chronic lung inflammation [165,186,187]. *Abcg1^−/−^* mice show massive infiltrates of lymphocytes and macrophages, high cholesterol levels, and increased expression of proinflammatory cytokines in the lungs by 8 months after birth. At the same time, inflammation in the lungs of *Abcg1^−/−^* mice is a secondary process that develops as a result of lipid accumulation [187].

Lipid loading of macrophages in *Abcg1^−/−^* mice was shown to be accompanied by increased production of proinflammatory IL-6, IL-1β, IL-1α, and IL-12 and a decrease in the anti-inflammatory IL-10 [185,187]. Elevated levels of matrix metalloproteinases (MMP)-8 and MMP-12, which are associated with destruction of the extracellular matrix, were also found in the lungs of *Abcg1^−/−^* mice [187]. The involvement of matrix metalloproteinases is well known in the pathogenesis of COPD because of their links to inflammation and airway remodeling [188,189].

Reverse cholesterol transport mediated by ABCA1 and ABCG1 is involved in the regulation of cholesterol content in macrophage plasma membranes [190]. Cholesterol is a key structural component of plasma membranes [191]. The unique properties of cholesterol ensure its participation in the lateral organization of the lipid bilayer, which enables the maintenance of optimum fluidity and creates the necessary conditions for the localization and function of membrane proteins [192]. Cholesterol is involved in the organization of lipid rafts, specialized microdomains of the plasma membrane, which act as dynamic platforms that ensure the assembly and functioning of many signaling pathways, including those associated with inflammation [193].

Changes in cholesterol content in the plasma membrane affect its biophysical properties as well as the localization and functional activity of membrane proteins [193]. The spatial arrangement of cholesterol molecules ensures its participation in the regulation of the function of transmembrane proteins through direct interaction of sterol with specific protein binding sites or through an indirect influence on the biophysical properties of the membrane [194]. It is assumed that proteins interacting with or binding cholesterol have characteristic amino acid sequences that play a role in this interaction. One such known sequence, the so-called cholesterol-binding amino acid domain (CRAC, cholesterol recognition/interaction amino acid consensus sequence), has been identified in proteins that interact with or are regulated by cholesterol [195,196]. The presence of CRAC or CARC amino acid sequences, in or near the transmembrane region, may suggest the possible involvement of cholesterol in the regulation of protein function.

Receptors whose function may change depending on cholesterol content include TLR4. TLR4 is considered a key participant in the initiation of inflammation in COPD because of its role in LPS detection [197,198,199,200,201]. TLR4 is thought to be able to bind directly to cholesterol through lipid–protein interactions, which has implications for the localization of the receptor in the plasma membrane and for its activation. TLR4 localizes in the lipid rafts of macrophage plasma membranes. Cholesterol content in lipid rafts affects the activation of the receptor signaling pathway. It was found that the transmembrane domain of TLR4 contains CRAC and CARC amino acid sequences that can provide a link between cholesterol and the regulation of receptor signal transduction [202]. Given that the CARC–CRAC–CARC domains in TLR4 are located close to the membrane, upstream of the TIR domain, this intracellular region of the receptor can specifically bind cholesterol [202].

The cholesterol content in lipid rafts is regulated by cholesterol transport from the plasma membrane to extracellular acceptors such as ApoA-I [203]. Thus, ApoA-I, ABCA1, and ABCG1, through their involvement in the export of cholesterol from the cell, can alter cellular cholesterol content, and thereby influence the biophysical properties of membranes and the structure of lipid rafts. Because of this, they are considered to be regulators of innate immunity.

Thus, ABCA1 and ABCG1, providing reverse cholesterol transport from macrophages, may be involved in the regulation of inflammation. Thus, reverse cholesterol transport is located at the crossroads of innate immunity and cholesterol metabolism.

Importantly, smoking impairs reverse cholesterol transport mediated by ABCA1 and ABCG1 [175,204,205]. This may lead to an increase in the number of macrophages overloaded with cholesterol, which contributes to their inflammatory activation [36,136]. In addition, the ABCA1 and ABCG1 transporters are an important link between COPD and atherosclerosis. Reduced lipid-exporting function of ABCA1 and ABCG1 in vascular wall macrophages is one of the key links to foam cell formation and progression of atherosclerosis [36,206]. Thus, ABCA1 and ABCG1, which are a link in reverse cholesterol transport, along with HDL demonstrate involvement in the pathogenesis of COPD and its comorbid links with atherosclerosis. At the same time, the mechanisms of such links are still a subject of research.

### 3.3. Other Anti-Inflammatory Mechanisms of HDL

In the bloodstream, HDL undergoes additional remodeling by lipid transport proteins (LCAT, cholesteryl ester transfer protein (CETP), and phospholipid transfer protein (PLTP)) as well as hepatic and endothelial lipases [163,207]. CETP belongs to a family of proteins including LBP and bactericidal permeability-increasing protein (BPI). Because of its lipid-transport function, CETP can participate in the transport of LPS between lipoproteins for further utilization in the liver (Figure 1) [208,209,210,211]. Kupffer cells take up most of the LPS and inactivate LPS by deacylation with acyloxyacyl hydrolase [212]. Despite a weaker ability to bind LPS compared with LBP or BPI, CETP is associated with resistance to sepsis [213,214]. Experiments with human CETP transgenic mice showed lower mortality after LPS administration compared with wild-type mice [213,215]. The pathway involving CETP is of interest because it represents a cross-talk mechanism between the reverse cholesterol transport and the innate immune system. In it, LPS and cholesterol share common transport and utilisation pathways [209]. This is also of interest when analyzing the links between COPD and atherosclerosis, for which impaired reverse cholesterol transport mechanisms and vascular inflammation are key links in development [209].

Studies show that CETP is associated with macrophage polarization in COPD. In huCETP mice, elastase-induced emphysema was characterized by an increase in the M2 phenotype of macrophages, indicating that CETP promoted M2 macrophage polarization [216]. CETP-M2 compared with WT-M2 showed increased transcription of ABCA1, which exported cholesterol from macrophages, decreased proinflammatory cytokine secretion, and increased IL-10 production, contributing to the anti-inflammatory M2 type response [216,217,218].

Attention to the role of CETP in immune response has increased because of the large number of infection-related deaths in the ILLUMINATE trial in which the CETP inhibitor torcetrapib was studied [219,220]. Meanwhile, torcetrapib showed good data to increase plasma HDL levels and reduce the development of atherosclerosis in a cholesterol-treated rabbit model [213,221].

Another mechanism of involvement in inflammation is related to the fact that HDL selectively prevents activation of type I interferon response genes when macrophages are exposed to LPS or the TLR4 agonist antibody. HDL is thought to inhibit TLR4 signaling in the pre-TRIF step, possibly by a mechanism involving the TRIF-related adaptor molecule (TRAM). HDL induces TRAM translocation into the intracellular compartment, thereby disrupting downstream TLR4 signaling. In this case, HDL deprives the plasma membrane of TRAM, a key adaptor molecule that activates TRIF in the endosomal compartments [222]. These findings are consistent with evidence that HDL regulates the cellular response to viral infection [222].

In addition, HDL mediates anti-inflammatory macrophage reprogramming via the transcriptional repressor ATF3. ATF3, induced by TLR activation, acts as part of an important negative feedback loop to limit inflammatory responses in macrophages. HDL uses this feedback mechanism to regulate inflammation [223].

Thus, HDL exhibits a variety of anti-inflammatory mechanisms, making it an important participant in the innate immune system. Importantly, HDL provides protection to LDL against oxidative free radical damage by reducing the production of proinflammatory oxidized lipids [224].

## 4. Proinflammatory Function of HDL

Interestingly, in addition to the known anti-inflammatory properties, HDL exhibits a dual role in inflammation (Figure 2) [225,226]. The proinflammatory effects of HDL are related to the fact that ApoA-1 can activate nuclear factor-kB and induce cytokine production in macrophages via TLR2, TLR4, CD14, and MyD88 [123].

Another mechanism is that HDL exerts proinflammatory effects on macrophages through passive cholesterol depletion and PKC-NF-κB /STAT1-IRF1 signaling [227]. HDL has been found to enhance toll-like receptor-induced signal transduction through activation of the PKC-NF-κB/STAT1–IRF1 axis, resulting in increased production of inflammatory cytokines [227].

Another proinflammatory effect of HDL arising from severe cholesterol depletion in macrophages is related to the endoplasmic reticulum (ER) stress response, which involves activation of the IRE1a (inositol-requiring enzyme 1a)/ASK1 (apoptosis signal-regulating kinase 1)/p38 MAPK (p38 mitogen-activated protein kinase) signaling axis [228].

It is important to note that inflammation can contribute to the transformation of HDL into a dysfunctional proinflammatory particle. In this case, HDL is characterized by a loss of the reverse cholesterol transport function and a loss of the ability to inhibit LDL oxidation [229,230]. The change in HDL function corresponds to some changes in HDL composition, characterized by the inclusion of oxidized phospholipids and proinflammatory proteins, such as serum amyloid A (SAA) [231]. SAA is synthesized mainly in the liver in response to infection and inflammation and is present in the plasma, where it is mainly bound to HDLs, affecting their function [232]. SAA impairs the anti-inflammatory properties of HDL [233]. The clinical significance of SAA is related to the fact that high SAA levels are associated with the frequency and severity of COPD exacerbations. SAA levels increase significantly during the acute phase of COPD exacerbations, making SAA a sensitive biomarker of exacerbation severity [234,235]. Moreover, in stable patients with COPD, SAA was independently associated with the phenotype of frequent exacerbations [234].

Thus, HDL shows both pro- and anti-inflammatory functions, which is of great importance and a prospect for further research.

## 5. Other Transport Functions of HDL

It has been shown that HDL can participate in intercellular communication mechanisms involving microRNA transport [236]. MicroRNAs are short non-coding RNAs consisting of 18–25 nucleotides, which take part in the post-transcriptional regulation of gene expression by targeting the coding and non-translated regions of target mRNAs.

MicroRNAs are present in the blood, where they are carried by exosomes and HDL [237,238].

HDL has been shown to be a carrier for hsa-miR-223 [236]. This microRNA is involved in the regulation of macrophage monocyte differentiation, neutrophil recruitment, and proinflammatory responses, so it may be involved in the pathogenesis of COPD [239]. By targeting Pknox1, miR-223 promotes alternative M2 activation of macrophages [240]. miR-223 negatively regulates progenitor proliferation, differentiation, and activation of granulocytes by targeting the Mef2c transcription factor [241]. Expression of miR-223 is significantly increased in lung tissue samples from COPD patients compared with smokers without airflow limitation [242]. MiR-223 is mainly expressed in myeloid cells and may play a role in innate immunity [239]. The involvement of miR-223 in inflammation has been shown to be related to its targeting of several links in the NF-κB pathway, such as PARP1, IKKa, TRAF6, CUL1, and TAB2 [239]. NF-κB activation is known to induce the expression of various proinflammatory genes and plays an important role in COPD [243]. It is assumed that miR-223 is a negative regulator of NF-κB signaling in both phagocytes and epithelial cells [244]. Overexpression of miR-223 reduces NLRP3 levels and IL-1β secretion and reduces airway inflammation in mice [245]. It has also been shown that miR-223 controls the expression and activity of HDAC2 in lung cells, which may alter the chemokine expression profile and influence the sensitivity to corticosteroids [239,246].

Thus, a protective role for miR-223 to reduce inflammation is suggested [239]. In addition, by targeting Mef2c, IGF-1R, TGFBR3, CDK2, and p53, miR-223 may participate in the regulation of cell proliferation, differentiation, viability, invasion, and death [239].

Thus, HDL may be involved in the regulation of inflammation in COPD by transporting microRNAs. In addition, the involvement of HDL in microRNA transport is of interest in cross-linkages with atherosclerosis. HDL has been shown to suppress the expression of intercellular adhesion molecule 1 (ICAM-1) through the transport of miR-223 into endothelial cells, which is another anti-inflammatory mechanism of HDL [247].

Another transport mechanism of interest in COPD is the transport of α1-antitrypsin (AAT) by HDL. AAT acts as an antagonist of neutrophil enzymes, providing the bulk of antiprotease activity in human serum [248]. AAT deficiency is one of the genetic causes of severe COPD [248]. HDL-associated AAT can be picked up via HDL receptors such as SR-B1, which can significantly increase the intracellular concentration of AAT [249]. HDL particles containing AAT may represent a functionally important type of HDL, protected from proteolytic damage and functional inactivation by elastase [250]. Moreover, AAT-enriched HDL particles show improved anti-inflammatory effects [251].

Thus, the transport functions of HDL play an important role in providing intercellular interactions and functions of the innate immune system. These data enhance the understanding of the role of HDL in the pathogenesis of COPD. At the same time, despite the fact that the available data suggest that there are links between HDL and the course of COPD, the details of these links are still largely elusive and are a promising topic of further research.

## 6. Conclusions

An analysis of the available data suggests the possible involvement of HDL in the pathogenesis of COPD, but the keys to understanding all of these connections are still not available to clinicians and researchers. A growing body of evidence suggests that the functions of HDL are not limited to simple cholesterol transport. HDLs are at the crossroads of lipid metabolism and the innate immune system. These are related to the role of cholesterol, which performs more than just structural functions, participating in the organization of the lipid bilayer of cell membranes. Through this organization, it influences the biophysical properties of membranes and the function of membrane proteins. The cholesterol load of macrophages is proinflammatory and impairs the participation of these cells in phagocytosis. In this regard, the regulation of cellular cholesterol content is a tool to control the inflammatory activation of macrophages. Impaired control of inflammatory activation of macrophages is one of the key links of COPD, which leads on the one hand to the increase in the number of these cells in the lungs and their production of proinflammatory factors, and on the other hand to impaired function of macrophages in phagocytosis, efferocytosis, and resolution of inflammation. In addition, apolipoproteins are direct participants in the innate immune system, owing to their ability to bind and neutralize LPS. The transport mechanisms in which LPS are involved are an additional link in a complex chain of processes associated not only with the movement of lipids, but also part of the protective mechanisms associated with the elimination of LPS. This seems important, given the differences in the composition of the lung microbiome in COPD, including the involvement of Gram-negative bacteria.

Thus, the innate immune system, which has a wide range of protective tools, relies on the transport potential of lipoproteins. Under these conditions, bacterial LPS in the bloodstream can bind to leukocytes, triggering immune mechanisms, or bind to lipoproteins that neutralize its biological activity [252]. These findings are of clinical interest when analyzing the pathogenesis of COPD. This widespread disease is characterized by chronic airway inflammation, progressive decline in lung function, increased severity of symptoms, cardiovascular comorbidity, and premature mortality. COPD is characterized by clinical heterogeneity, which includes variability in the severity of symptoms; differences in the rate of decline in lung function, frequency, and severity of exacerbations; development of emphysema; and other characteristics. Disturbances in the structure of airway bacteria microbiota are well known in these patients and are closely associated with the severity of exacerbations. These data emphasize the role of the innate immune system and disorders of its regulation mechanisms in COPD.

It should be noted that the clinical heterogeneity of COPD may complicate the interpretation of data in studies on the role of HDL. The differences in the results of the studies may be related to the clinical heterogeneity of COPD patients, which is due to the individual characteristics of pathophysiological mechanisms and lipid metabolism.

Thus, HDL demonstrates an important role in inflammation, which is associated with the intersection of the lipid-transport function of HDL and mechanisms of the innate immune system. At the same time, the clinical significance of HDL in COPD requires further study. The anti-inflammatory role of HDL in the clinically heterogeneous course of COPD and the analysis of the involvement of HDL in the mechanisms of the comorbid course of COPD and atherosclerosis are promising research directions for future studies. In addition, the transport function of HDL for microRNAs is of interest. These and other data will improve the current knowledge on the role of HDLs in inflammation and their possible involvement in the pathogenesis of COPD.

## Data Availability

Not applicable.

## References

[1] Adeloye D., Song P., Zhu Y., Campbell H., Sheikh A., Rudan I. (2022). Global, regional, and national prevalence of, and risk factors for, chronic obstructive pulmonary disease (COPD) in 2019: A systematic review and modelling analysis. Lancet Respir. Med..

[2] Blanco I., Diego I., Bueno P., Casas-Maldonado F., Miravitlles M. (2019). Geographic distribution of COPD prevalence in the world displayed by Geographic Information System maps. Eur. Respir. J..

[3] Søgaard M., Madsen M., Løkke A., Hilberg O., Sørensen H.T., Thomsen R.W. (2016). Incidence and outcomes of patients hospitalized with COPD exacerbation with and without pneumonia. Int. J. Chronic Obstr. Pulm. Dis..

[4] Westbroek L.F., Klijnsma M., Salome P., Sekhuis L.M., Rolink E., Korsmit E., Kerstjens H.A.M., Group L.C.C.P.S. (2020). Reducing the Number of Hospitalization Days for COPD: Setting up a Transmural-Care Pathway. Int. J. Chronic Obstruct. Pulm. Dis..

[5] Chronic Obstructive Pulmonary Disease (COPD). https://www.who.int/news-room/fact-sheets/detail/chronic-obstructive-pulmonary-disease-(copd).

[6] García Castillo E., Alonso Pérez T., Ancochea J., Pastor Sanz M.T., Almagro P., Martínez-Camblor P., Miravitlles M., Rodríguez-Carballeira M., Navarro A., Lamprecht B. (2020). Mortality prediction in chronic obstructive pulmonary disease comparing the GOLD 2015 and GOLD 2019 staging: A pooled analysis of individual patient data. ERJ Open Res..

[7] Hogg J.C. (2004). Pathophysiology of airflow limitation in chronic obstructive pulmonary disease. Lancet.

[8] (2022). Global Strategy for Prevention, Diagnosis and Management of COPD: 2022 Report. https://goldcopd.org/2022-gold-reports-2/.

[9] Marciniuk D.D., Goodridge D., Hernandez P., Rocker G., Balter M., Bailey P., Ford G., Bourbeau J., O’Donnell D.E., Maltais F. (2011). Managing dyspnea in patients with advanced chronic obstructive pulmonary disease: A Canadian Thoracic Society clinical practice guideline. Can. Respir. J..

[10] Miravitlles M., Menezes A., López Varela M.V., Casas A., Ugalde L., Ramirez-Venegas A., Mendoza L., López A., Wehrmeister F.C., Surmont F. (2018). Prevalence and impact of respiratory symptoms in a population of patients with COPD in Latin America: The LASSYC observational study. Respir. Med..

[11] Gephine S., Mucci P., Grosbois J.-M., Maltais F., Saey D. (2021). Physical Frailty in COPD Patients with Chronic Respiratory Failure. Int. J. Chronic Obstr. Pulm. Dis..

[12] Bernard S., LeBlanc P., Whittom F., Carrier G., Jobin J., Belleau R., Maltais F. (1998). Peripheral muscle weakness in patients with chronic obstructive pulmonary disease. Am. J. Respir. Crit. Care Med..

[13] Laniado-Laborín R. (2009). Smoking and chronic obstructive pulmonary disease (COPD). Parallel epidemics of the 21 century. Int. J. Environ. Res. Public Health.

[14] Marsh S., Aldington S., Shirtcliffe P., Weatherall M., Beasley R. (2006). Smoking and COPD: What really are the risks?. Eur. Respir. J..

[15] Franciosi L., Postma D.S., van den Berge M., Govorukhina N., Horvatovich P.L., Fusetti F., Poolman B., Lodewijk M.E., Timens W., Bischoff R. (2014). Susceptibility to COPD: Differential proteomic profiling after acute smoking. PLoS ONE.

[16] Leem A.Y., Park B., Kim Y.S., Chang J., Won S., Jung J.Y. (2019). Longitudinal decline in lung function: A community-based cohort study in Korea. Sci. Rep..

[17] Safitri W., Martini S., Artanti K.D., Li C.-Y. (2021). Smoking from a Younger Age Is the Dominant Factor in the Incidence of Chronic Obstructive Pulmonary Disease: Case-Control Study. Int. J. Environ. Res. Public Health.

[18] Chen H., Liu X., Gao X., Lv Y., Zhou L., Shi J., Wei W., Huang J., Deng L., Wang Z. (2021). Epidemiological evidence relating risk factors to chronic obstructive pulmonary disease in China: A systematic review and meta-analysis. PLoS ONE.

[19] Jiang X.-Q., Mei X.-D., Feng D. (2016). Air pollution and chronic airway diseases: What should people know and do?. J. Thorac. Dis..

[20] Duan R.-R., Hao K., Yang T. (2020). Air pollution and chronic obstructive pulmonary disease. Chronic Dis. Transl. Med..

[21] Denden S., Khelil A.H., Knani J., Lakhdar R., Perrin P., Lefranc G., Chibani J.B. (2010). Alpha-1 antitrypsin gene polymorphism in Chronic Obstructive Pulmonary Disease (COPD). Genet. Mol. Biol..

[22] Angelis N., Porpodis K., Zarogoulidis P., Spyratos D., Kioumis I., Papaiwannou A., Pitsiou G., Tsakiridis K., Mpakas A., Arikas S. (2014). Airway inflammation in chronic obstructive pulmonary disease. J. Thorac. Dis..

[23] Sutherland E.R., Martin R.J. (2003). Airway inflammation in chronic obstructive pulmonary disease: Comparisons with asthma. J. Allergy Clin. Immunol..

[24] Kotlyarov S. (2022). Involvement of the Innate Immune System in the Pathogenesis of Chronic Obstructive Pulmonary Disease. Int. J. Mol. Sci..

[25] Vlahos R., Bozinovski S. (2014). Role of alveolar macrophages in chronic obstructive pulmonary disease. Front. Immunol..

[26] Jasper A.E., McIver W.J., Sapey E., Walton G.M. (2019). Understanding the role of neutrophils in chronic inflammatory airway disease. F1000Research.

[27] Shaykhiev R., Crystal R.G. (2013). Innate immunity and chronic obstructive pulmonary disease: A mini-review. Gerontology.

[28] Monsó E. (2017). Microbiome in chronic obstructive pulmonary disease. Ann. Transl. Med..

[29] Wang Z., Bafadhel M., Haldar K., Spivak A., Mayhew D., Miller B.E., Tal-Singer R., Johnston S.L., Ramsheh M.Y., Barer M.R. (2016). Lung microbiome dynamics in COPD exacerbations. Eur. Respir. J..

[30] Kerkhof M., Voorham J., Dorinsky P., Cabrera C., Darken P., Kocks J.W., Sadatsafavi M., Sin D.D., Carter V., Price D.B. (2020). Association between COPD exacerbations and lung function decline during maintenance therapy. Thorax.

[31] Makris D., Moschandreas J., Damianaki A., Ntaoukakis E., Siafakas N.M., Milic Emili J., Tzanakis N. (2007). Exacerbations and lung function decline in COPD: New insights in current and ex-smokers. Respir. Med..

[32] Groenewegen K.H., Postma D.S., Hop W.C., Wielders P.L., Schlösser N.J., Wouters E.F. (2008). Increased systemic inflammation is a risk factor for COPD exacerbations. Chest.

[33] Oudijk E.J.D., Lammers J.W.J., Koenderman L. (2003). Systemic inflammation in chronic obstructive pulmonary disease. Eur. Respir. J..

[34] Barnes P.J., Celli B.R. (2009). Systemic manifestations and comorbidities of COPD. Eur. Respir. J..

[35] Chen H., Li Z., Dong L., Wu Y., Shen H., Chen Z. (2019). Lipid metabolism in chronic obstructive pulmonary disease. Int. J. Chronic Obstr. Pulm. Dis..

[36] Kotlyarov S., Kotlyarova A. (2021). The Role of ABC Transporters in Lipid Metabolism and the Comorbid Course of Chronic Obstructive Pulmonary Disease and Atherosclerosis. Int. J. Mol. Sci..

[37] Naik D., Joshi A., Paul T.V., Thomas N. (2014). Chronic obstructive pulmonary disease and the metabolic syndrome: Consequences of a dual threat. Indian J. Endocrinol. Metab..

[38] Mortensen M.B., Nordestgaard B.G. (2020). Elevated LDL cholesterol and increased risk of myocardial infarction and atherosclerotic cardiovascular disease in individuals aged 70–100 years: A contemporary primary prevention cohort. Lancet.

[39] Linton M.F., Yancey P.G., Davies S.S., Jerome W.G., Linton E.F., Song W.L., Doran A.C., Vickers K.C., Feingold K.R., Anawalt B., Boyce A., Chrousos G., de Herder W.W., Dhatariya K., Dungan K., Hershman J.M., Hofland J., Kalra S. (2000). The Role of Lipids and Lipoproteins in Atherosclerosis. Endotext.

[40] Ference B.A., Ginsberg H.N., Graham I., Ray K.K., Packard C.J., Bruckert E., Hegele R.A., Krauss R.M., Raal F.J., Schunkert H. (2017). Low-density lipoproteins cause atherosclerotic cardiovascular disease. 1. Evidence from genetic, epidemiologic, and clinical studies. A consensus statement from the European Atherosclerosis Society Consensus Panel. Eur. Heart J..

[41] Soppert J., Lehrke M., Marx N., Jankowski J., Noels H. (2020). Lipoproteins and lipids in cardiovascular disease: From mechanistic insights to therapeutic targeting. Adv. Drug Deliv. Rev..

[42] Atar D., Jukema J.W., Molemans B., Taub P.R., Goto S., Mach F., CerezoOlmos C., Underberg J., Keech A., Tokgözoğlu L. (2021). New cardiovascular prevention guidelines: How to optimally manage dyslipidaemia and cardiovascular risk in 2021 in patients needing secondary prevention?. Atherosclerosis.

[43] Zafirova-Ivanovska B., Stojkovikj J., Dokikj D., Anastasova S., Debresliovska A., Zejnel S., Stojkovikj D. (2016). The Level of Cholesterol in COPD Patients with Severe and Very Severe Stage of the Disease. Open Access Maced. J. Med. Sci..

[44] Jungck D., Zickfeld M.I., Albuscheit T., Brandenburger E., Knobloch J., Koch A. (2017). HDL serum levels are significantly lower in patients with COPD than in never-smokers. Eur. Respir. J..

[45] Rafie S., Moitra S., Brashier B.B. (2018). Association between the Serum Metabolic Profile and Lung Function in Chronic Obstructive Pulmonary Disease. Turk. Thorac. J..

[46] Burkart K.M., Manichaikul A., Wilk J.B., Ahmed F.S., Burke G.L., Enright P., Hansel N.N., Haynes D., Heckbert S.R., Hoffman E.A. (2014). *APOM* and high-density lipoprotein cholesterol are associated with lung function and per cent emphysema. Eur. Respir. J..

[47] Shi Y., Zhang J., Huang Y. (2021). Prediction of cardiovascular risk in patients with chronic obstructive pulmonary disease: A study of the National Health and Nutrition Examination Survey database. BMC Cardiovasc. Disord..

[48] Tantucci C., Modina D. (2012). Lung function decline in COPD. Int. J. Chronic Obstr. Pulm. Dis..

[49] Marott J.L., Ingebrigtsen T.S., Çolak Y., Vestbo J., Lange P. (2020). Lung Function Trajectories Leading to Chronic Obstructive Pulmonary Disease as Predictors of Exacerbations and Mortality. Am. J. Respir. Crit. Care Med..

[50] Oelsner E., Balte P., Schwartz J.E., Burkart K.M., Cassano P., Jacobs D.R., Kalhan R., Kronmal R., Loehr L.R., O’Connor G.T. (2016). LATE-BREAKING ABSTRACT: High density lipoprotein cholesterol (HDL-C) and longitudinal lung function in six United States (US) cohorts. Eur. Respir. J..

[51] Park J.H., Mun S., Choi D.P., Lee J.Y., Kim H.C. (2017). Association between high-density lipoprotein cholesterol level and pulmonary function in healthy Korean adolescents: The JS high school study. BMC Pulm. Med..

[52] Huerta-Ramírez S., Paniagua-Pérez A., Castro-Serna D., Ledesma-Velázquez A., Rubio-Guerra A., Vargas-Ayala G. (2018). Effect of the components of the metabolic syndrome on pulmonary function. The unexpected role of high-density lipoprotein cholesterol. Cir. Cir..

[53] Reed R.M., Hashmi S., Eberlein M., Iacono A., Netzer G., DeFilippis A., Girgis R.E., Toth P.P., Scharf S., Jones S. (2011). Impact of lung transplantation on serum lipids in COPD. Respir. Med..

[54] Reed R.M., Iacono A., DeFilippis A., Eberlein M., Girgis R.E., Jones S. (2011). Advanced chronic obstructive pulmonary disease is associated with high levels of high-density lipoprotein cholesterol. J. Heart Lung Transplant..

[55] Huang Y., Jiang B., Miao X., Ma J., Wang J., Ding K., Chen X., Hu Q., Fu F., Zeng T. (2020). The Relationship of Lymphocyte to High-Density Lipoprotein Ratio with Pulmonary Function in COPD. Int. J. Chronic Obstr. Pulm. Dis..

[56] Li H., Liu Y., Wang L., Shen T., Du W., Liu Z., Chen R., Hu M. (2016). High apolipoprotein M serum levels correlate with chronic obstructive pulmonary disease. Lipids Health Dis..

[57] Can U., Yerlikaya F.H., Yosunkaya S. (2015). Role of oxidative stress and serum lipid levels in stable chronic obstructive pulmonary disease. J. Chin. Med. Assoc..

[58] Xuan L., Han F., Gong L., Lv Y., Wan Z., Liu H., Zhang D., Jia Y., Yang S., Ren L. (2018). Association between chronic obstructive pulmonary disease and serum lipid levels: A meta-analysis. Lipids Health Dis..

[59] Burkart K.M., Ahmed F.S., Watson K., Hoffman E.A., Burke G.L., Barr R.G. (2010). Association between high density lipoproteins (hdl) cholesterol and ct percent emphysema. The mesa lung study. B37. Chronic Obstructive Pulmonary Disease Pathogenesis I.

[60] Ogawa E., Nakano Y., Ohara T., Muro S., Hirai T., Sato S., Sakai H., Tsukino M., Kinose D., Nishioka M. (2009). Body mass index in male patients with COPD: Correlation with low attenuation areas on CT. Thorax.

[61] Coxson H.O., Chan I.H., Mayo J.R., Hlynsky J., Nakano Y., Birmingham C.L. (2004). Early emphysema in patients with anorexia nervosa. Am. J. Respir. Crit. Care Med..

[62] Takahashi S., Betsuyaku T. (2015). The chronic obstructive pulmonary disease comorbidity spectrum in Japan differs from that in western countries. Respir. Investig..

[63] Viniol C., Vogelmeier C.F. (2018). Exacerbations of COPD. Eur. Respir. Rev..

[64] Oelsner E., Burkart K., Jacobs D., Loehr L., Kalhan R., O’Connor G., Tsai M., White W., Kronmal R., Folsom A. (2015). LATE-BREAKING ABSTRACT: High density lipoprotein cholesterol (HDL) and severe chronic lung disease (CLD) exacerbations in the general population. Eur. Respir. J..

[65] Madsen C.M., Varbo A., Tybjærg-Hansen A., Frikke-Schmidt R., Nordestgaard B.G. (2017). U-shaped relationship of HDL and risk of infectious disease: Two prospective population-based cohort studies. Eur. Heart J..

[66] García-Olmos L., Alberquilla Á., Ayala V., García-Sagredo P., Morales L., Carmona M., de Tena-Dávila M.J., Pascual M., Muñoz A., Salvador C.H. (2013). Comorbidity in patients with chronic obstructive pulmonary disease in family practice: A cross sectional study. BMC Fam. Pract..

[67] Fragoso E., André S., Boleo-Tomé J.P., Areias V., Munhá J., Cardoso J. (2016). Understanding COPD: A vision on phenotypes, comorbidities and treatment approach. Rev. Port. Pneumol..

[68] Terzikhan N., Lahousse L., Verhamme K.M.C., Franco O.H., Ikram A.M., Stricker B.H., Brusselle G.G. (2018). COPD is associated with an increased risk of peripheral artery disease and mortality. ERJ Open Res..

[69] Aisanov Z., Khaltaev N. (2020). Management of cardiovascular comorbidities in chronic obstructive pulmonary disease patients. J. Thorac. Dis..

[70] Cavaillès A., Brinchault-Rabin G., Dixmier A., Goupil F., Gut-Gobert C., Marchand-Adam S., Meurice J.-C., Morel H., Person-Tacnet C., Leroyer C. (2013). Comorbidities of COPD. Eur. Respir. Rev..

[71] Enriquez J.R., Parikh S.V., Selzer F., Jacobs A.K., Marroquin O., Mulukutla S., Srinivas V., Holper E.M. (2011). Increased adverse events after percutaneous coronary intervention in patients with COPD: Insights from the National Heart, Lung, and Blood Institute dynamic registry. Chest.

[72] Zhou W., Li C.-L., Cao J., Feng J. (2020). Metabolic syndrome prevalence in patients with obstructive sleep apnea syndrome and chronic obstructive pulmonary disease: Relationship with systemic inflammation. Clin. Respir. J..

[73] Kuklisova Z., Tkacova R., Joppa P., Wouters E., Sastry M. (2017). Severity of nocturnal hypoxia and daytime hypercapnia predicts CPAP failure in patients with COPD and obstructive sleep apnea overlap syndrome. Sleep Med..

[74] Khatri S.B., Ioachimescu O.C. (2016). The intersection of obstructive lung disease and sleep apnea. Cleve. Clin. J. Med..

[75] Choi H.S., Rhee C.K., Park Y.B., Yoo K.H., Lim S.Y. (2019). Metabolic Syndrome in Early Chronic Obstructive Pulmonary Disease: Gender Differences and Impact on Exacerbation and Medical Costs. Int. J. Chronic Obstr. Pulm. Dis..

[76] Sahoo K.C., Subhankar S., Mohanta P.C., Jagaty S.K., Dutta P., Pothal S. (2022). Prevalence of metabolic syndrome in chronic obstructive pulmonary disease and its correlation with severity of disease. J. Fam. Med. Prim. Care.

[77] Geslain-Biquez C., Vol S., Tichet J., Caradec A., D’Hour A., Balkau B. (2003). The metabolic syndrome in smokers. The D.E.S.I.R. study. Diabetes Metab..

[78] Sun Y., Milne S., Jaw J.E., Yang C.X., Xu F., Li X., Obeidat M.e., Sin D.D. (2019). BMI is associated with FEV1 decline in chronic obstructive pulmonary disease: A meta-analysis of clinical trials. Respir. Res..

[79] Divo M.J., Cabrera C., Casanova C., Marin J.M., Pinto-Plata V.M., de-Torres J.P., Zulueta J., Zagaceta J., Sanchez-Salcedo P., Berto J. (2014). Comorbidity Distribution, Clinical Expression and Survival in COPD Patients with Different Body Mass Index. Chronic Obstr. Pulm. Dis. J. COPD Found..

[80] Grigsby M.R., Siddharthan T., Pollard S.L., Chowdhury M., Rubinstein A., Miranda J.J., Bernabe-Ortiz A., Alam D., Kirenga B., Jones R. (2019). Low Body Mass Index Is Associated with Higher Odds of COPD and Lower Lung Function in Low- and Middle-Income Countries. COPD J. Chronic Obstr. Pulm. Dis..

[81] Kim E.K., Singh D., Park J.H., Park Y.B., Kim S.I., Park B., Park J., Kim J.H., Kim M.A., Lee J.H. (2020). Impact of Body Mass Index Change on the Prognosis of Chronic Obstructive Pulmonary Disease. Respiration.

[82] Wada H., Ikeda A., Maruyama K., Yamagishi K., Barnes P.J., Tanigawa T., Tamakoshi A., Iso H. (2021). Low BMI and weight loss aggravate COPD mortality in men, findings from a large prospective cohort: The JACC study. Sci. Rep..

[83] Park H.J., Cho J.H., Kim H.J., Park J.-Y., Lee H.S., Byun M.K. (2019). The effect of low body mass index on the development of chronic obstructive pulmonary disease and mortality. J. Intern. Med..

[84] Guo Y., Zhang T., Wang Z., Yu F., Xu Q., Guo W., Wu C., He J. (2016). Body mass index and mortality in chronic obstructive pulmonary disease: A dose-response meta-analysis. Medicine.

[85] Iyer A.S., Dransfield M.T. (2018). The “Obesity Paradox” in Chronic Obstructive Pulmonary Disease: Can It Be Resolved?. Ann. Am. Thorac. Soc..

[86] Wu T.D., Ejike C.O., Wise R.A., McCormack M.C., Brigham E.P. (2019). Investigation of the Obesity Paradox in Chronic Obstructive Pulmonary Disease, According to Smoking Status, in the United States. Am. J. Epidemiol..

[87] Collins P.F., Stratton R.J., Kurukulaaratchy R., Elia M. (2010). S163 The ‘Obesity Paradox’ in chronic obstructive pulmonary disease. Thorax.

[88] Martinez-Aguilar E., Orbe J., Fernández-Montero A., Fernández-Alonso S., Rodríguez J.A., Fernández-Alonso L., Páramo J.A., Roncal C. (2017). Reduced high-density lipoprotein cholesterol: A valuable, independent prognostic marker in peripheral arterial disease. J. Vasc. Surg..

[89] Kanbay M., Solak Y., Unal H.U., Kurt Y.G., Gok M., Cetinkaya H., Karaman M., Oguz Y., Eyileten T., Vural A. (2014). Monocyte count/HDL cholesterol ratio and cardiovascular events in patients with chronic kidney disease. Int. Urol. Nephrol..

[90] Kundi H., Kiziltunc E., Cetin M., Cicekcioglu H., Cetin Z.G., Cicek G., Ornek E. (2016). Association of monocyte/HDL-C ratio with SYNTAX scores in patients with stable coronary artery disease. Herz.

[91] Bolayir A., Gokce S.F., Cigdem B., Bolayir H.A., Yildiz O.K., Bolayir E., Topaktas S.A. (2018). Monocyte/high-density lipoprotein ratio predicts the mortality in ischemic stroke patients. Neurol. Neurochir. Pol..

[92] Yakar H.I., Kanbay A. (2020). Could monocyte level/HDL cholesterol ratio predict cardiovascular diseases in patients with COPD?. Niger. J. Clin. Pract..

[93] Jiang M., Yang J., Zou H., Li M., Sun W., Kong X. (2022). Monocyte-to-high-density lipoprotein-cholesterol ratio (MHR) and the risk of all-cause and cardiovascular mortality: A nationwide cohort study in the United States. Lipids Health Dis..

[94] Acikgoz N., Kurtoğlu E., Yagmur J., Kapicioglu Y., Cansel M., Ermis N. (2018). Elevated Monocyte to High-Density Lipoprotein Cholesterol Ratio and Endothelial Dysfunction in Behçet Disease. Angiology.

[95] Yılmaz M., Kayançiçek H. (2018). A New Inflammatory Marker: Elevated Monocyte to HDL Cholesterol Ratio Associated with Smoking. J. Clin. Med..

[96] Young R.P., Hopkins R.J. (2013). Update on the potential role of statins in chronic obstructive pulmonary disease and its co-morbidities. Expert Rev. Respir. Med..

[97] Young R., Hopkins R., Eaton T. (2009). Pharmacological actions of statins: Potential utility in COPD. Eur. Respir. Rev..

[98] Walsh A., Perrem L., Khashan A.S., Henry M.T., Ni Chroinin M. (2019). Statins versus placebo for people with chronic obstructive pulmonary disease. Cochrane Database Syst. Rev..

[99] Rezk N.A., Elewa A. (2013). Anti inflammatory effects of statin in COPD. Egypt. J. Chest Dis. Tuberc..

[100] Damkjær M., Håkansson K., Kallemose T., Ulrik C.S., Godtfredsen N. (2021). Statins in High-Risk Chronic Obstructive Pulmonary Disease Outpatients: No Impact on Time to First Exacerbation and All-Cause Mortality—The STATUETTE Cohort Study. Int. J. Chronic Obstr. Pulm. Dis..

[101] Raymakers A.J., Sadatsafavi M., Sin D.D., De Vera M.A., Lynd L.D. (2017). The impact of statin drug use on all-cause mortality in patients with COPD: A population-based cohort study. Chest.

[102] Cao C., Wu Y., Xu Z., Lv D., Zhang C., Lai T., Li W., Shen H. (2015). The effect of statins on chronic obstructive pulmonary disease exacerbation and mortality: A systematic review and meta-analysis of observational research. Sci. Rep..

[103] Ingebrigtsen T.S., Marott J.L., Nordestgaard B.G., Lange P., Hallas J., Vestbo J. (2015). Statin use and exacerbations in individuals with chronic obstructive pulmonary disease. Thorax.

[104] Yayan J., Bald M., Franke K.-J. (2021). No Independent Influence of Statins on the Chronic Obstructive Pulmonary Disease Exacerbation Rate: A Cohort Observation Study Over 10 Years. Int. J. Gen. Med..

[105] Smith M.C., Ashdown H.F., Sheppard J.P., Butler C.C., Bankhead C. (2021). Statin prescription in patients with chronic obstructive pulmonary disease and risk of exacerbations: A retrospective cohort study in the Clinical Practice Research Datalink. BMJ Open.

[106] Yeh J.-J., Lin C.-L., Hsu C.Y., Shae Z., Kao C.-H. (2019). Associations between statins and coronary artery disease and stroke risks in patients with asthma–chronic obstructive pulmonary disease overlap syndrome: A time-dependent regression study. Atherosclerosis.

[107] Nazir S., Jankowski V., Bender G., Zewinger S., Rye K.-A., van der Vorst E.P.C. (2020). Interaction between high-density lipoproteins and inflammation: Function matters more than concentration!. Adv. Drug Deliv. Rev..

[108] Navab M., Reddy S.T., Van Lenten B.J., Fogelman A.M. (2011). HDL and cardiovascular disease: Atherogenic and atheroprotective mechanisms. Nat. Rev. Cardiol..

[109] Bandeali S., Farmer J. (2012). High-density lipoprotein and atherosclerosis: The role of antioxidant activity. Curr. Atheroscler. Rep..

[110] Sallese A., Suzuki T., McCarthy C., Bridges J., Filuta A., Arumugam P., Shima K., Ma Y., Wessendarp M., Black D. (2017). Targeting cholesterol homeostasis in lung diseases. Sci. Rep..

[111] Remmerie A., Scott C.L. (2018). Macrophages and lipid metabolism. Cell. Immunol..

[112] Carey B., Trapnell B.C. (2010). The molecular basis of pulmonary alveolar proteinosis. Clin. Immunol..

[113] Tall A.R., Yvan-Charvet L. (2015). Cholesterol, inflammation and innate immunity. Nat. Rev. Immunol..

[114] Pian M.S., Dobbs L.G. (1997). Lipoprotein-stimulated surfactant secretion in alveolar type II cells: Mediation by heterotrimeric G proteins. Am. J. Physiol..

[115] Kolleck I., Schlame M., Fechner H., Looman A.C., Wissel H., Rüstow B. (1999). HDL is the major source of vitamin E for type II pneumocytes. Free Radic. Biol. Med..

[116] Han R. (2010). Plasma lipoproteins are important components of the immune system. Microbiol. Immunol..

[117] Kaji H. (2013). High-density lipoproteins and the immune system. J. Lipids.

[118] Shibata N., Glass C.K. (2009). Regulation of macrophage function in inflammation and atherosclerosis. J. Lipid Res..

[119] Zannis V.I., Cole F.S., Jackson C.L., Kurnit D.M., Karathanasis S.K. (1985). Distribution of apolipoprotein A-I, C-II, C-III, and E mRNA in fetal human tissues. Time-dependent induction of apolipoprotein E mRNA by cultures of human monocyte-macrophages. Biochemistry.

[120] Kingwell B.A., Chapman M.J., Kontush A., Miller N.E. (2014). HDL-targeted therapies: Progress, failures and future. Nat. Rev. Drug Discov..

[121] Zannis V.I., Fotakis P., Koukos G., Kardassis D., Ehnholm C., Jauhiainen M., Chroni A., von Eckardstein A., Kardassis D. (2015). HDL Biogenesis, Remodeling, and Catabolism. High Density Lipoproteins: From Biological Understanding to Clinical Exploitation.

[122] Zhao G.-J., Yin K., Fu Y.-C., Tang C.-K. (2012). The interaction of ApoA-I and ABCA1 triggers signal transduction pathways to mediate efflux of cellular lipids. Mol. Med..

[123] Smoak K.A., Aloor J.J., Madenspacher J., Merrick B.A., Collins J.B., Zhu X., Cavigiolio G., Oda M.N., Parks J.S., Fessler M.B. (2010). Myeloid differentiation primary response protein 88 couples reverse cholesterol transport to inflammation. Cell Metab..

[124] Wang N., Chen W., Linsel-Nitschke P., Martinez L.O., Agerholm-Larsen B., Silver D.L., Tall A.R. (2003). A PEST sequence in ABCA1 regulates degradation by calpain protease and stabilization of ABCA1 by apoA-I. J. Clin. Investig..

[125] Cooke A.L., Morris J., Melchior J.T., Street S.E., Jerome W.G., Huang R., Herr A.B., Smith L.E., Segrest J.P., Remaley A.T. (2018). A thumbwheel mechanism for APOA1 activation of LCAT activity in HDL. J. Lipid Res..

[126] Sorci-Thomas M.G., Bhat S., Thomas M.J. (2009). Activation of lecithin:cholesterol acyltransferase by HDL ApoA-I central helices. Clin. Lipidol..

[127] Cooke A., Melchior J.T., Morris J.C., Huang R., Jerome W.G., Davidson W.S. (2016). Abstract 541: The Molecular Interaction of Apolipoprotein A-I Containing High Density Lipoproteins with Lecithin: Cholesterol Acyl Transferase. Arterioscler. Thromb. Vasc. Biol..

[128] Casteleijn M.G., Parkkila P., Viitala T., Koivuniemi A. (2018). Interaction of lecithin:cholesterol acyltransferase with lipid surfaces and apolipoprotein A-I-derived peptides. J. Lipid Res..

[129] Sankaranarayanan S., Oram J.F., Asztalos B.F., Vaughan A.M., Lund-Katz S., Adorni M.P., Phillips M.C., Rothblat G.H. (2009). Effects of acceptor composition and mechanism of ABCG1-mediated cellular free cholesterol efflux. J. Lipid Res..

[130] Yao X., Gordon E.M., Figueroa D.M., Barochia A.V., Levine S.J. (2016). Emerging Roles of Apolipoprotein E and Apolipoprotein A-I in the Pathogenesis and Treatment of Lung Disease. Am. J. Respir. Cell Mol. Biol..

[131] Gordon E.M., Figueroa D.M., Barochia A.V., Yao X., Levine S.J. (2016). High-density Lipoproteins and Apolipoprotein A-I: Potential New Players in the Prevention and Treatment of Lung Disease. Front. Pharmacol..

[132] Wang W., Xu H., Shi Y., Nandedkar S., Zhang H., Gao H., Feroah T., Weihrauch D., Schulte M.L., Jones D.W. (2010). Genetic deletion of apolipoprotein A-I increases airway hyperresponsiveness, inflammation, and collagen deposition in the lung. J. Lipid Res..

[133] Provost P.R., Boucher E., Tremblay Y. (2009). Apolipoprotein A-I, A-II, C-II, and H expression in the developing lung and sex difference in surfactant lipids. J. Endocrinol..

[134] Kim C., Lee J.M., Park S.W., Kim K.S., Lee M.W., Paik S., Jang A.S., Kim D.J., Uh S., Kim Y. (2016). Attenuation of Cigarette Smoke-Induced Emphysema in Mice by Apolipoprotein A-1 Overexpression. Am. J. Respir. Cell Mol. Biol..

[135] Slagter S.N., van Vliet-Ostaptchouk J.V., Vonk J.M., Boezen H.M., Dullaart R.P.F., Kobold A.C.M., Feskens E.J., van Beek A.P., van der Klauw M.M., Wolffenbuttel B.H.R. (2013). Associations between smoking, components of metabolic syndrome and lipoprotein particle size. BMC Med..

[136] Jubinville É., Talbot M., Bérubé J.-C., Hamel-Auger M., Maranda-Robitaille M., Beaulieu M.-J., Aubin S., Paré M.-È., Kallend D.G., Arsenault B. (2017). Interplay between cigarette smoking and pulmonary reverse lipid transport. Eur. Respir. J..

[137] Barochia A.V., Kaler M., Cuento R.A., Gordon E.M., Weir N.A., Sampson M., Fontana J.R., MacDonald S., Moss J., Manganiello V. (2015). Serum apolipoprotein A-I and large high-density lipoprotein particles are positively correlated with FEV1 in atopic asthma. Am. J. Respir. Crit. Care Med..

[138] Kotlyarov S.N., Kotlyarova A.A. (2021). Role of lipid metabolism and systemic inflammation in the development of atherosclerosis in animal models. IP Pavlov. Russ. Med. Biol. Her..

[139] Catapano A.L., Pirillo A., Bonacina F., Norata G.D. (2014). HDL in innate and adaptive immunity. Cardiovasc. Res..

[140] Emancipator K., Csako G., Elin R.J. (1992). In vitro inactivation of bacterial endotoxin by human lipoproteins and apolipoproteins. Infect. Immun..

[141] Ma J., Liao X.L., Lou B., Wu M.P. (2004). Role of apolipoprotein A-I in protecting against endotoxin toxicity. Acta Biochim. Biophys. Sin..

[142] Jiao Y.L., Wu M.P. (2008). Apolipoprotein A-I diminishes acute lung injury and sepsis in mice induced by lipoteichoic acid. Cytokine.

[143] Henning M.F., Herlax V., Bakás L. (2011). Contribution of the C-terminal end of apolipoprotein AI to neutralization of lipopolysaccharide endotoxic effect. Innate Immun..

[144] Van Linthout S., Spillmann F., Graiani G., Miteva K., Peng J., Van Craeyveld E., Meloni M., Tölle M., Escher F., Subasigüller A. (2011). Down-regulation of endothelial TLR4 signalling after apo A-I gene transfer contributes to improved survival in an experimental model of lipopolysaccharide-induced inflammation. J. Mol. Med..

[145] Sharifov O.F., Xu X., Gaggar A., Grizzle W.E., Mishra V.K., Honavar J., Litovsky S.H., Palgunachari M.N., White C.R., Anantharamaiah G.M. (2013). Anti-inflammatory mechanisms of apolipoprotein A-I mimetic peptide in acute respiratory distress syndrome secondary to sepsis. PLoS ONE.

[146] Brandenburg K., Jürgens G., Andrä J., Lindner B., Koch M.H.J., Blume A., Garidel P. (2002). Biophysical characterization of the interaction of high-density lipoprotein (HDL) with endotoxins. Eur. J. Biochem..

[147] Georgila K., Vyrla D., Drakos E. (2019). Apolipoprotein A-I (ApoA-I), Immunity, Inflammation and Cancer. Cancers.

[148] Levine D.M., Parker T.S., Donnelly T.M., Walsh A., Rubin A.L. (1993). In vivo protection against endotoxin by plasma high density lipoprotein. Proc. Natl. Acad. Sci. USA.

[149] Wurfel M.M., Kunitake S.T., Lichenstein H., Kane J.P., Wright S.D. (1994). Lipopolysaccharide (LPS)-binding protein is carried on lipoproteins and acts as a cofactor in the neutralization of LPS. J. Exp. Med..

[150] Dietrich M.A., Adamek M., Bilińska B., Hejmej A., Steinhagen D., Ciereszko A. (2014). Characterization, expression and antibacterial properties of apolipoproteins A from carp (*Cyprinus carpio* L.) seminal plasma. Fish Shellfish. Immunol..

[151] Dietrich M.A., Nynca J., Adamek M., Steinhagen D., Karol H., Ciereszko A. (2015). Expression of apolipoprotein A-I and A-II in rainbow trout reproductive tract and their possible role in antibacterial defence. Fish Shellfish. Immunol..

[152] Karan S., Mohapatra A., Sahoo P.K., Garg L.C., Dixit A. (2020). Structural-functional characterization of recombinant Apolipoprotein A-I fromLabeo rohitademonstrates heat-resistant antimicrobial activity. Appl. Microbiol. Biotechnol..

[153] Wang W., Qu Q., Chen J. (2019). Identification, expression analysis, and antibacterial activity of Apolipoprotein A-I from amphioxus (*Branchiostoma belcheri*). Comp. Biochem. Physiol. Part B Biochem. Mol. Biol..

[154] Biedzka-Sarek M., Metso J., Kateifides A., Meri T., Jokiranta T.S., Muszyński A., Radziejewska-Lebrecht J., Zannis V., Skurnik M., Jauhiainen M. (2011). Apolipoprotein A-I Exerts Bactericidal Activity against Yersinia enterocolitica Serotype O:3*. J. Biol. Chem..

[155] Singh I.P., Chopra A.K., Coppenhaver D.H., Ananatharamaiah G.M., Baron S. (1999). Lipoproteins account for part of the broad non-specific antiviral activity of human serum. Antivir. Res..

[156] Tang C., Houston B.A., Storey C., LeBoeuf R.C. (2016). Both STAT3 activation and cholesterol efflux contribute to the anti-inflammatory effect of apoA-I/ABCA1 interaction in macrophages. J. Lipid Res..

[157] Cheng A.M., Handa P., Tateya S., Schwartz J., Tang C., Mitra P., Oram J.F., Chait A., Kim F. (2012). Apolipoprotein A-I attenuates palmitate-mediated NF-κB activation by reducing Toll-like receptor-4 recruitment into lipid rafts. PLoS ONE.

[158] Zhang M., Li L., Xie W., Wu J.F., Yao F., Tan Y.L., Xia X.D., Liu X.Y., Liu D., Lan G. (2016). Apolipoprotein A-1 binding protein promotes macrophage cholesterol efflux by facilitating apolipoprotein A-1 binding to ABCA1 and preventing ABCA1 degradation. Atherosclerosis.

[159] Low H., Mukhamedova N., Capettini L.d.S.A., Xia Y., Carmichael I., Cody S.H., Huynh K., Ditiatkovski M., Ohkawa R., Bukrinsky M. (2020). Cholesterol Efflux-Independent Modification of Lipid Rafts by AIBP (Apolipoprotein A-I Binding Protein). Arterioscler. Thromb. Vasc. Biol..

[160] Zhang M., Zhao G.J., Yin K., Xia X.D., Gong D., Zhao Z.W., Chen L.Y., Zheng X.L., Tang X.E., Tang C.K. (2018). Apolipoprotein A-1 Binding Protein Inhibits Inflammatory Signaling Pathways by Binding to Apolipoprotein A-1 in THP-1 Macrophages. Circ. J..

[161] Choi S.H., Wallace A.M., Schneider D.A., Burg E., Kim J., Alekseeva E., Ubags N.D., Cool C.D., Fang L., Suratt B.T. (2018). AIBP augments cholesterol efflux from alveolar macrophages to surfactant and reduces acute lung inflammation. JCI Insight.

[162] Furlaneto C.J., Ribeiro F.P., Hatanaka E., Souza G.M., Cassatella M.A., Campa A. (2002). Apolipoproteins A-I and A-II downregulate neutrophil functions. Lipids.

[163] Meilhac O., Tanaka S., Couret D. (2020). High-Density Lipoproteins Are Bug Scavengers. Biomolecules.

[164] Jacobo-Albavera L., Domínguez-Pérez M., Medina-Leyte D.J., González-Garrido A., Villarreal-Molina T. (2021). The Role of the ATP-Binding Cassette A1 (ABCA1) in Human Disease. Int. J. Mol. Sci..

[165] Chai A.B., Ammit A.J., Gelissen I.C. (2017). Examining the role of ABC lipid transporters in pulmonary lipid homeostasis and inflammation. Respir. Res..

[166] Yvan-Charvet L., Ranalletta M., Wang N., Han S., Terasaka N., Li R., Welch C., Tall A.R. (2007). Combined deficiency of ABCA1 and ABCG1 promotes foam cell accumulation and accelerates atherosclerosis in mice. J. Clin. Investig..

[167] Nagao K., Tomioka M., Ueda K. (2011). Function and regulation of ABCA1—Membrane meso-domain organization and reorganization. FEBS J..

[168] Li Y., Schwabe R.F., DeVries-Seimon T., Yao P.M., Gerbod-Giannone M.C., Tall A.R., Davis R.J., Flavell R., Brenner D.A., Tabas I. (2005). Free cholesterol-loaded macrophages are an abundant source of tumor necrosis factor-alpha and interleukin-6: Model of NF-kappaB- and map kinase-dependent inflammation in advanced atherosclerosis. J. Biol. Chem..

[169] Gerbod-Giannone M.-C., Li Y., Holleboom A., Han S., Hsu L.-C., Tabas I., Tall A.R. (2006). TNFα induces ABCA1 through NF-κB in macrophages and in phagocytes ingesting apoptotic cells. Proc. Natl. Acad. Sci. USA.

[170] Iwamoto N., Abe-Dohmae S., Sato R., Yokoyama S. (2006). ABCA7 expression is regulated by cellular cholesterol through the SREBP2 pathway and associated with phagocytosis. J. Lipid Res..

[171] Abe-Dohmae S., Ikeda Y., Matsuo M., Hayashi M., Okuhira K.-i., Ueda K., Yokoyama S. (2004). Human ABCA7 Supports Apolipoprotein-mediated Release of Cellular Cholesterol and Phospholipid to Generate High Density Lipoprotein*. J. Biol. Chem..

[172] Tanaka N., Abe-Dohmae S., Iwamoto N., Fitzgerald M.L., Yokoyama S. (2010). Helical apolipoproteins of high-density lipoprotein enhance phagocytosis by stabilizing ATP-binding cassette transporter A7 [S]. J. Lipid Res..

[173] Bates S.R., Tao J.-Q., Collins H.L., Francone O.L., Rothblat G.H. (2005). Pulmonary abnormalities due to ABCA1 deficiency in mice. Am. J. Physiol.-Lung Cell. Mol. Physiol..

[174] McNeish J., Aiello R.J., Guyot D., Turi T., Gabel C., Aldinger C., Hoppe K.L., Roach M.L., Royer L.J., de Wet J. (2000). High density lipoprotein deficiency and foam cell accumulation in mice with targeted disruption of ATP-binding cassette transporter-1. Proc. Natl. Acad. Sci. USA.

[175] Kotlyarov S., Kotlyarova A. (2021). Bioinformatic Analysis of ABCA1 Gene Expression in Smoking and Chronic Obstructive Pulmonary Disease. Membranes.

[176] Yu Z., Jin J., Wang Y., Sun J. (2017). High density lipoprotein promoting proliferation and migration of type II alveolar epithelial cells during inflammation state. Lipids Health Dis..

[177] Wang N., Lan D., Chen W., Matsuura F., Tall A.R. (2004). ATP-binding cassette transporters G1 and G4 mediate cellular cholesterol efflux to high-density lipoproteins. Proc. Natl. Acad. Sci. USA.

[178] Bensinger S.J., Bradley M.N., Joseph S.B., Zelcer N., Janssen E.M., Hausner M.A., Shih R., Parks J.S., Edwards P.A., Jamieson B.D. (2008). LXR signaling couples sterol metabolism to proliferation in the acquired immune response. Cell.

[179] Out R., Hoekstra M., Habets K., Meurs I., de Waard V., Hildebrand R.B., Wang Y., Chimini G., Kuiper J., Van Berkel T.J. (2008). Combined deletion of macrophage ABCA1 and ABCG1 leads to massive lipid accumulation in tissue macrophages and distinct atherosclerosis at relatively low plasma cholesterol levels. Arterioscler. Thromb. Vasc. Biol..

[180] Vaughan A.M., Oram J.F. (2006). ABCA1 and ABCG1 or ABCG4 act sequentially to remove cellular cholesterol and generate cholesterol-rich HDL. J. Lipid Res..

[181] Gelissen I.C., Harris M., Rye K.A., Quinn C., Brown A.J., Kockx M., Cartland S., Packianathan M., Kritharides L., Jessup W. (2006). ABCA1 and ABCG1 synergize to mediate cholesterol export to apoA-I. Arterioscler. Thromb. Vasc. Biol..

[182] Jessup W., Gelissen I.C., Gaus K., Kritharides L. (2006). Roles of ATP binding cassette transporters A1 and G1, scavenger receptor BI and membrane lipid domains in cholesterol export from macrophages. Curr. Opin. Lipidol..

[183] Oram J.F., Vaughan A.M. (2006). ATP-Binding cassette cholesterol transporters and cardiovascular disease. Circ. Res..

[184] Seres L., Cserepes J., Elkind N.B., Törocsik D., Nagy L., Sarkadi B., Homolya L. (2008). Functional ABCG1 expression induces apoptosis in macrophages and other cell types. Biochim. Biophys. Acta.

[185] Wojcik A.J., Skaflen M.D., Srinivasan S., Hedrick C.C. (2008). A critical role for ABCG1 in macrophage inflammation and lung homeostasis. J. Immunol..

[186] Baldan A., Gonen A., Choung C., Que X., Marquart T.J., Hernandez I., Bjorkhem I., Ford D.A., Witztum J.L., Tarling E.J. (2014). ABCG1 is required for pulmonary B-1 B cell and natural antibody homeostasis. J. Immunol..

[187] Baldán Á., Gomes A.V., Ping P., Edwards P.A. (2008). Loss of ABCG1 Results in Chronic Pulmonary Inflammation. J. Immunol..

[188] Churg A., Zhou S., Wright J.L. (2012). Matrix metalloproteinases in COPD. Eur. Respir. J..

[189] Sng J.J., Prazakova S., Thomas P.S., Herbert C. (2017). MMP-8, MMP-9 and Neutrophil Elastase in Peripheral Blood and Exhaled Breath Condensate in COPD. COPD J. Chronic Obstr. Pulm. Dis..

[190] Draper D.W., Madenspacher J.H., Dixon D., King D.H., Remaley A.T., Fessler M.B. (2010). ATP-binding cassette transporter G1 deficiency dysregulates host defense in the lung. Am. J. Respir. Crit. Care Med..

[191] Yang S.-T., Kreutzberger A.J.B., Lee J., Kiessling V., Tamm L.K. (2016). The role of cholesterol in membrane fusion. Chem. Phys. Lipids.

[192] Devaux P.F., Morris R. (2004). Transmembrane Asymmetry and Lateral Domains in Biological Membranes. Traffic.

[193] Song Y., Kenworthy A.K., Sanders C.R. (2014). Cholesterol as a co-solvent and a ligand for membrane proteins. Protein Sci..

[194] Fantini J., Epand R.M., Barrantes F.J., Rosenhouse-Dantsker A., Bukiya A.N. (2019). Cholesterol-Recognition Motifs in Membrane Proteins. Direct Mechanisms in Cholesterol Modulation of Protein Function.

[195] Fantini J., Barrantes F. (2013). How cholesterol interacts with membrane proteins: An exploration of cholesterol-binding sites including CRAC, CARC, and tilted domains. Front. Physiol..

[196] Sharpe L.J., Rao G., Jones P.M., Glancey E., Aleidi S.M., George A.M., Brown A.J., Gelissen I.C. (2015). Cholesterol sensing by the ABCG1 lipid transporter: Requirement of a CRAC motif in the final transmembrane domain. Biochim. Biophys. Acta (BBA)—Mol. Cell Biol. Lipids.

[197] Sidletskaya K., Vitkina T., Denisenko Y. (2020). The Role of Toll-Like Receptors 2 and 4 in the Pathogenesis of Chronic Obstructive Pulmonary Disease. Int. J. Chronic Obstr. Pulm. Dis..

[198] Beutler B., Du X., Poltorak A. (2001). Identification of Toll-like receptor 4 (Tlr4) as the sole conduit for LPS signal transduction: Genetic and evolutionary studies. J. Endotoxin Res..

[199] Wong S.W., Kwon M.-J., Choi A.M.K., Kim H.-P., Nakahira K., Hwang D.H. (2009). Fatty acids modulate Toll-like receptor 4 activation through regulation of receptor dimerization and recruitment into lipid rafts in a reactive oxygen species-dependent manner. J. Biol. Chem..

[200] Sarir H., Mortaz E., Karimi K., Kraneveld A.D., Rahman I., Caldenhoven E., Nijkamp F.P., Folkerts G. (2009). Cigarette smoke regulates the expression of TLR4 and IL-8 production by human macrophages. J. Inflamm..

[201] Zhang X., Shan P., Jiang G., Cohn L., Lee P.J. (2006). Toll-like receptor 4 deficiency causes pulmonary emphysema. J. Clin. Investig..

[202] Ruysschaert J.M., Lonez C. (2015). Role of lipid microdomains in TLR-mediated signalling. Biochim. Biophys. Acta.

[203] Murphy A.J., Woollard K.J., Hoang A., Mukhamedova N., Stirzaker R.A., McCormick S.P., Remaley A.T., Sviridov D., Chin-Dusting J. (2008). High-density lipoprotein reduces the human monocyte inflammatory response. Arterioscler. Thromb. Vasc. Biol..

[204] He P., Gelissen I.C., Ammit A.J. (2020). Regulation of ATP binding cassette transporter A1 (ABCA1) expression: Cholesterol-dependent and—Independent signaling pathways with relevance to inflammatory lung disease. Respir. Res..

[205] Sonett J., Goldklang M., Sklepkiewicz P., Gerber A., Trischler J., Zelonina T., Westerterp M., Lemaître V., Okada Y., D’Armiento J. (2018). A critical role for ABC transporters in persistent lung inflammation in the development of emphysema after smoke exposure. FASEB J..

[206] Matsuo M. (2022). ABCA1 and ABCG1 as potential therapeutic targets for the prevention of atherosclerosis. J. Pharmacol. Sci..

[207] Lewis G.F., Rader D.J. (2005). New insights into the regulation of HDL metabolism and reverse cholesterol transport. Circ. Res..

[208] Blauw L.L., Wang Y., Willems van Dijk K., Rensen P.C.N. (2020). A Novel Role for CETP as Immunological Gatekeeper: Raising HDL to Cure Sepsis?. Trends Endocrinol. Metab..

[209] Azzam K.M., Fessler M.B. (2012). Crosstalk between reverse cholesterol transport and innate immunity. Trends Endocrinol. Metab..

[210] Topchiy E., Cirstea M., Kong H.J., Boyd J.H., Wang Y., Russell J.A., Walley K.R. (2016). Lipopolysaccharide Is Cleared from the Circulation by Hepatocytes via the Low Density Lipoprotein Receptor. PLoS ONE.

[211] Cai L., Ji A., de Beer F.C., Tannock L.R., van der Westhuyzen D.R. (2008). SR-BI protects against endotoxemia in mice through its roles in glucocorticoid production and hepatic clearance. J. Clin. Investig..

[212] Munford R.S., Weiss J.P., Lu M. (2020). Biochemical transformation of bacterial lipopolysaccharides by acyloxyacyl hydrolase reduces host injury and promotes recovery. J. Biol. Chem..

[213] Shrestha S., Wu B.J., Guiney L., Barter P.J., Rye K.-A. (2018). Cholesteryl ester transfer protein and its inhibitors. J. Lipid Res..

[214] Trinder M., Genga K.R., Kong H.J., Blauw L.L., Lo C., Li X., Cirstea M., Wang Y., Rensen P.C.N., Russell J.A. (2019). Cholesteryl Ester Transfer Protein Influences High-Density Lipoprotein Levels and Survival in Sepsis. Am. J. Respir. Crit. Care Med..

[215] Cazita P.M., Barbeiro D.F., Moretti A.I., Quintão E.C., Soriano F.G. (2008). Human cholesteryl ester transfer protein expression enhances the mouse survival rate in an experimental systemic inflammation model: A novel role for CETP. Shock.

[216] Santana K.G., Righetti R.F., Breda C.N.d.S., Domínguez-Amorocho O.A., Ramalho T., Dantas F.E.B., Nunes V.S., Tibério I.d.F.L.C., Soriano F.G., Câmara N.O.S. (2021). Cholesterol-Ester Transfer Protein Alters M1 and M2 Macrophage Polarization and Worsens Experimental Elastase-Induced Pulmonary Emphysema. Front. Immunol..

[217] Ma L., Dong F., Zaid M., Kumar A., Zha X. (2012). ABCA1 protein enhances Toll-like receptor 4 (TLR4)-stimulated interleukin-10 (IL-10) secretion through protein kinase A (PKA) activation. J. Biol. Chem..

[218] Tall A.R. (2008). Cholesterol efflux pathways and other potential mechanisms involved in the athero-protective effect of high density lipoproteins. J. Intern. Med..

[219] Barter P.J., Caulfield M., Eriksson M., Grundy S.M., Kastelein J.J., Komajda M., Lopez-Sendon J., Mosca L., Tardif J.C., Waters D.D. (2007). Effects of torcetrapib in patients at high risk for coronary events. N. Engl. J. Med..

[220] Di Bartolo B.A., Duong M., Nicholls S.J. (2016). Clinical trials with cholesteryl ester transfer protein inhibitors. Curr. Opin. Lipidol.

[221] Morehouse L.A., Sugarman E.D., Bourassa P.A., Sand T.M., Zimetti F., Gao F., Rothblat G.H., Milici A.J. (2007). Inhibition of CETP activity by torcetrapib reduces susceptibility to diet-induced atherosclerosis in New Zealand White rabbits. J. Lipid Res..

[222] Suzuki M., Pritchard D.K., Becker L., Hoofnagle A.N., Tanimura N., Bammler T.K., Beyer R.P., Bumgarner R., Vaisar T., Beer M.C.d. (2010). High-Density Lipoprotein Suppresses the Type I Interferon Response, a Family of Potent Antiviral Immunoregulators, in Macrophages Challenged With Lipopolysaccharide. Circulation.

[223] De Nardo D., Labzin L.I., Kono H., Seki R., Schmidt S.V., Beyer M., Xu D., Zimmer S., Lahrmann C., Schildberg F.A. (2014). High-density lipoprotein mediates anti-inflammatory reprogramming of macrophages via the transcriptional regulator ATF3. Nat. Immunol..

[224] Brites F., Martin M., Guillas I., Kontush A. (2017). Antioxidative activity of high-density lipoprotein (HDL): Mechanistic insights into potential clinical benefit. BBA Clin..

[225] Rohatgi A., Westerterp M., Eckardstein A.v., Remaley A., Rye K.-A. (2021). HDL in the 21st Century: A Multifunctional Roadmap for Future HDL Research. Circulation.

[226] Ansell B.J., Fonarow G.C., Fogelman A.M. (2006). High-density lipoprotein: Is it always atheroprotective?. Curr. Atheroscler. Rep..

[227] van der Vorst E.P.C., Theodorou K., Wu Y., Hoeksema M.A., Goossens P., Bursill C.A., Aliyev T., Huitema L.F.A., Tas S.W., Wolfs I.M.J. (2017). High-Density Lipoproteins Exert Pro-inflammatory Effects on Macrophages via Passive Cholesterol Depletion and PKC-NF-κB/STAT1-IRF1 Signaling. Cell Metab..

[228] Fotakis P., Kothari V., Thomas D.G., Westerterp M., Molusky M.M., Altin E., Abramowicz S., Wang N., He Y., Heinecke J.W. (2019). Anti-Inflammatory Effects of HDL (High-Density Lipoprotein) in Macrophages Predominate Over Proinflammatory Effects in Atherosclerotic Plaques. Arterioscler. Thromb. Vasc. Biol..

[229] Van Lenten B.J., Hama S.Y., de Beer F.C., Stafforini D.M., McIntyre T.M., Prescott S.M., La Du B.N., Fogelman A.M., Navab M. (1995). Anti-inflammatory HDL becomes pro-inflammatory during the acute phase response. Loss of protective effect of HDL against LDL oxidation in aortic wall cell cocultures. J. Clin. Investig..

[230] Navab M., Reddy S.T., Van Lenten B.J., Anantharamaiah G.M., Fogelman A.M. (2009). The role of dysfunctional HDL in atherosclerosis. J. Lipid Res..

[231] G H.B., Rao V.S., Kakkar V.V. (2011). Friend Turns Foe: Transformation of Anti-Inflammatory HDL to Proinflammatory HDL during Acute-Phase Response. Cholesterol.

[232] Uhlar C.M., Whitehead A.S. (1999). Serum amyloid A, the major vertebrate acute-phase reactant. Eur. J. Biochem..

[233] Han C.Y., Tang C., Guevara M.E., Wei H., Wietecha T., Shao B., Subramanian S., Omer M., Wang S., O’Brien K.D. (2016). Serum amyloid A impairs the antiinflammatory properties of HDL. J. Clin. Investig..

[234] Zhao D., Abbasi A., Rossiter H.B., Su X., Liu H., Pi Y., Sang L., Zhong W., Yang Q., Guo X. (2020). Serum Amyloid A in Stable COPD Patients is Associated with the Frequent Exacerbator Phenotype. Int. J. Chronic Obstr. Pulm. Dis..

[235] Bozinovski S., Hutchinson A., Thompson M., Macgregor L., Black J., Giannakis E., Karlsson A.S., Silvestrini R., Smallwood D., Vlahos R. (2008). Serum amyloid a is a biomarker of acute exacerbations of chronic obstructive pulmonary disease. Am. J. Respir. Crit. Care Med..

[236] Vickers K.C., Palmisano B.T., Shoucri B.M., Shamburek R.D., Remaley A.T. (2011). MicroRNAs are transported in plasma and delivered to recipient cells by high-density lipoproteins. Nat. Cell Biol..

[237] Chen X., Ba Y., Ma L., Cai X., Yin Y., Wang K., Guo J., Zhang Y., Chen J., Guo X. (2008). Characterization of microRNAs in serum: A novel class of biomarkers for diagnosis of cancer and other diseases. Cell Res..

[238] Vickers K.C., Remaley A.T. (2012). Lipid-based carriers of microRNAs and intercellular communication. Curr. Opin. Lipidol..

[239] Roffel M.P., Bracke K.R., Heijink I.H., Maes T. (2020). miR-223: A Key Regulator in the Innate Immune Response in Asthma and COPD. Front. Med..

[240] Haneklaus M., Gerlic M., O’Neill L.A.J., Masters S.L. (2013). miR-223: Infection, inflammation and cancer. J. Intern. Med..

[241] Johnnidis J.B., Harris M.H., Wheeler R.T., Stehling-Sun S., Lam M.H., Kirak O., Brummelkamp T.R., Fleming M.D., Camargo F.D. (2008). Regulation of progenitor cell proliferation and granulocyte function by microRNA-223. Nature.

[242] Ezzie M.E., Crawford M., Cho J.-H., Orellana R., Zhang S., Gelinas R., Batte K., Yu L., Nuovo G., Galas D. (2012). Gene expression networks in COPD: microRNA and mRNA regulation. Thorax.

[243] Schuliga M. (2015). NF-kappaB Signaling in Chronic Inflammatory Airway Disease. Biomolecules.

[244] Zhou W., Pal A.S., Hsu A.Y.-H., Gurol T., Zhu X., Wirbisky-Hershberger S.E., Freeman J.L., Kasinski A.L., Deng Q. (2018). MicroRNA-223 Suppresses the Canonical NF-κB Pathway in Basal Keratinocytes to Dampen Neutrophilic Inflammation. Cell Rep..

[245] Xu W., Wang Y., Ma Y., Yang J. (2020). MiR-223 plays a protecting role in neutrophilic asthmatic mice through the inhibition of NLRP3 inflammasome. Respir. Res..

[246] Leuenberger C., Schuoler C., Bye H., Mignan C., Rechsteiner T., Hillinger S., Opitz I., Marsland B., Faiz A., Hiemstra P.S. (2016). MicroRNA-223 controls the expression of histone deacetylase 2: A novel axis in COPD. J. Mol. Med..

[247] Tabet F., Vickers K.C., Cuesta Torres L.F., Wiese C.B., Shoucri B.M., Lambert G., Catherinet C., Prado-Lourenco L., Levin M.G., Thacker S. (2014). HDL-transferred microRNA-223 regulates ICAM-1 expression in endothelial cells. Nat. Commun..

[248] da Costa C.H., Noronha Filho A.J., Marques e Silva R.M.F., da Cruz T.F., de Oliveira Monteiro V., Pio M., Rufino R.L. (2019). Alpha 1-antitrypsin deficiency in patients with chronic obstructive pulmonary disease patients: Is systematic screening necessary?. BMC Res. Notes.

[249] Moreno J.-A., Ortega-Gomez A., Rubio-Navarro A., Louedec L., Ho-Tin-Noé B., Caligiuri G., Nicoletti A., Levoye A., Plantier L., Meilhac O. (2014). High-Density Lipoproteins Potentiate α1-Antitrypsin Therapy in Elastase-Induced Pulmonary Emphysema. Am. J. Respir. Cell Mol. Biol..

[250] Gordon S.M., Sviridov D., Sakurai T., Freeman L., Remaley A.T. (2016). Abstract 29: Alpha-1-antitrypsin Protects High Density Lipoprotein From Functional Inactivation by Elastase. Arterioscler. Thromb. Vasc. Biol..

[251] Segal L., Lewis E.C. (2022). The lipid ties of α1-antitrypsin: Structural and functional aspects. Cell Immunol..

[252] Kitchens R.L., Wolfbauer G., Albers J.J., Munford R.S. (1999). Plasma lipoproteins promote the release of bacterial lipopolysaccharide from the monocyte cell surface. J. Biol. Chem..

